# Soluble Forms and Ligands of the Receptor for Advanced Glycation End-Products in Patients with Acute Respiratory Distress Syndrome: An Observational Prospective Study

**DOI:** 10.1371/journal.pone.0135857

**Published:** 2015-08-14

**Authors:** Matthieu Jabaudon, Raiko Blondonnet, Laurence Roszyk, Bruno Pereira, Renaud Guérin, Sébastien Perbet, Sophie Cayot, Damien Bouvier, Loic Blanchon, Vincent Sapin, Jean-Michel Constantin

**Affiliations:** 1 CHU Clermont-Ferrand, Intensive Care Unit, Department of Anesthesiology, Critical Care and Perioperative Medicine, Estaing Hospital, Clermont-Ferrand, France; 2 Clermont Université, Université d'Auvergne, Clermont-Ferrand, France; 3 CHU Clermont-Ferrand, Department of Medical Biochemistry and Molecular Biology, Estaing University Hospital, Clermont-Ferrand, France; 4 CHU Clermont-Ferrand, Department of Clinical Research and Innovation (DRCI), Clermont-Ferrand, France; Medical University of South Carolina, UNITED STATES

## Abstract

**Background:**

The main soluble form of the receptor for advanced glycation end-products (sRAGE) is elevated during acute respiratory distress syndrome (ARDS). However other RAGE isoforms and multiple ligands have been poorly reported in the clinical setting, and their respective contribution to RAGE activation during ARDS remains unclear. Our goal was therefore to describe main RAGE isoforms and ligands levels during ARDS.

**Methods:**

30 ARDS patients and 30 mechanically ventilated controls were prospectively included in this monocenter observational study. Arterial, superior vena cava and alveolar fluid levels of sRAGE, endogenous-secretory RAGE (esRAGE), high mobility group box-1 protein (HMGB1), S100A12 and advanced glycation end-products (AGEs) were measured in duplicate ELISA on day 0, day 3 and day 6. In patients with ARDS, baseline lung morphology was assessed with computed tomography.

**Results:**

ARDS patients had higher arterial, central venous and alveolar levels of sRAGE, HMGB1 and S100A12, but lower levels of esRAGE and AGEs, than controls. Baseline arterial sRAGE, HMGB1 and S100A12 were correlated with nonfocal ARDS (AUC 0.79, 0.65 and 0.63, respectively). Baseline arterial sRAGE, esRAGE, S100A12 and AGEs were associated with severity as assessed by PaO_2_/FiO_2_.

**Conclusions:**

This is the first kinetics study of levels of RAGE main isoforms and ligands during ARDS. Elevated sRAGE, HMGB1 and S100A12, with decreased esRAGE and AGEs, were found to distinguish patients with ARDS from those without. Our findings should prompt future studies aimed at elucidating RAGE/HMGB1/S100A12 axis involvement in ARDS.

**Trial Registration:**

clinicaltrials.gov Identifier: NCT01270295.

## Introduction

Acute respiratory distress syndrome (ARDS) is a major cause of acute respiratory failure in critically ill patients[1–3]. ARDS is characterized by diffuse lung epithelial and endothelial injury leading to increased permeability alveolar edema[4]. Few interventions have proved beneficial in ARDS[5–7] and pharmacological approaches remain limited. The magnitude of damage to the alveolar type (AT) I cell is a major determinant of ARDS severity and outcomes[8]. RAGE, the receptor for advanced glycation end-products, plays a pivotal role in ARDS pathogenesis[9–12]. RAGE is a transmembrane receptor of the immunoglobulin superfamily that is primarily located on the basal surface of AT I cells[9]. Its activation modulates cell signaling, culminating in a sustained inflammatory response leading to the activation of nuclear transcription factor NF-κB[13]. RAGE can be measured in biological fluids as soluble RAGE (sRAGE, comprising the extracellular domain of RAGE, and produced through the cleavage of full-length RAGE by proteinases)[14, 15] and endogenous secretory RAGE (esRAGE, produced after alternative splicing)[16]. RAGE is downregulated in most tissues under physiologic conditions, but its expression and activation are upregulated where ligands accumulate[13]. RAGE binds diverse ligands including advanced glycation end-products (AGEs), β-amyloid peptides and damage-associated molecular patterns, e.g. S100 proteins and *high mobility group box-1 protein* (HMGB1)[17–20]. Multiple pathways may be involved in RAGE activation[21], and RAGE isoforms may act as decoy receptors that prevent activation of transmembrane RAGE by ligands[22]. As RAGE isoforms and ligands have been poorly reported to date, we designed our study to report their levels during ARDS.

## Materials and Methods

### Ethics statements

Our institutional review board (*Comité de Protection des Personnes Sud-Est VI*) approved the study protocol. All participants, or their next-of-kin, provided written consent to participate.

### Setting

This was a monocenter, prospective observational study. Patients were screened and enrolled between February 2011 and January 2013 in the general intensive care unit (ICU) at Estaing University Hospital, Clermont-Ferrand, France, after the protocol was registered on clinicaltrials.gov (NCT01270295).

Patient management was based on our ICU standard protocols. Mechanical ventilation strategy (including weaning), sepsis management, and sedation were based on available guidelines[5, 23]. Lung-protective ventilation was applied; a tidal volume of 6 ml/kg (predicted) body weight and a plateau pressure under 30 cmH_2_O were targeted[5].

### Study patients

Thirty consecutive patients with acute lung injury (ALI)/ARDS were identified based on the American-European Consensus Conference definition[24] and included within 24 hours of disease onset. Thirty age- and sex-matched patients under mechanical ventilation but without ALI/ARDS served as controls. Patients were ineligible if: they were pregnant; <18 years old; they had a history of acute exacerbation of diabetes, dialysis for end-stage kidney disease, Alzheimer’s disease, amyloidosis or evolutive solid neoplasm.

### Clinical data

Clinical and biological data were obtained for all living patients at all timepoints. According to our institutional protocol, a lung computed tomography (CT) scan was performed on day 0 in ARDS patients[25]. Two independent radiologists performed the qualitative CT analysis according to the “CT-scan ARDS study group” criteria[26]. Three patterns of loss-of-aeration distribution were identified: focal, diffuse, patchy. Nonfocal morphology was noted for patients with diffuse or patchy patterns[27]. Clinical outcome was recorded until day 28.

### Biologic sample collection, processing and measurements

In both groups, arterial blood and central venous blood from the superior vena cava were simultaneously sampled on day 0 (at inclusion (D0H0) and 4 hours later (D0H4)), day 3 (D3) and day 6 (D6) after inclusion; undiluted bronchoalveolar fluid was collected simultaneously[28]. Blood and alveolar samples were immediately centrifuged at 1,000xg for 15 minutes; supernatant was kept frozen at -80°C until analysis. Biological markers were measured in duplicate using commercially available sandwich enzyme-linked immunosorbent assay (ELISA) kits: sRAGE (Human sRAGE Quantikine Kit, R&D Systems, Minneapolis, MN, USA), esRAGE (esRAGE Kit, B-Bridge International, Mountain View, CA, USA), HMGB1 (HMGB1 Kit, IBL International, Hamburg, Germany), S100A12 (CircuLex S100A12/EN-RAGE Kit, MBL International, Nagano, Japan), AGEs (OxiSelect AGEs Kit, Cell Biolabs, San Diego, CA, USA). AGEs kit detects and quantifies N-**ε**-carboxymethyl-lysine and pentosidine, the two main AGEs.

### Study outcomes

The primary outcome was the difference between groups in baseline plasma levels of biological markers. Secondary objectives were: to describe RAGE soluble forms (sRAGE, esRAGE) and ligands (HMGB1, S100A12, AGEs) pulmonary and plasma levels during the first 6 days after ARDS onset, as compared with measurements in controls; to report venous-to-arterial differences and alveolar-to-arterial ratios in sRAGE, esRAGE, HMGB1, S100A12 and AGEs levels; to test their correlation with CT-scan lung morphology and ARDS severity; to test their prognostic values in ARDS.

### Statistical analyses

Sample size was difficult to estimate because RAGE isoforms and ligands levels have been poorly investigated to date in the clinical setting of ARDS. Few data are available on sRAGE levels variability in ICU patients[10–12], with standard deviations around 2000 pg/ml. When considering type I error alpha and statistical power of 5% (bilateral) and 80%, respectively, enrolling 30 patients in each group would allow the detection of a 1500 pg/ml difference in (baseline arterial) sRAGE levels, which corresponds to an effect size of 0.75[29]. Categorical data were expressed as numbers and percentages, and quantitative data as mean and standard deviation (SD) or median and interquartile range (IQR) according to statistical distribution. Log-transformation was proposed to reach normal distribution. Student t-test or Mann-Whitney test were used to compare quantitative parameters. Proportions were compared using Chi^2^ or Fisher’s exact tests. Spearman correlation coefficient was calculated to study the relation between quantitative variables. Correlations between biological markers and severity (e.g., PaO_2_/FiO_2_ ratio) were tested using both univariate and multivariate analyses. Repeated data were analyzed using mixed models evaluating group, time and their interaction effects, taking into account between- and within-subject variability. Receiver-operating characteristic (ROC) curves were computed to determine which parameter better distinguished nonfocal from focal ARDS. Areas under the curve (AUC) were calculated and reported with 95% confidence interval (CI)(e.g., for sensitivity and specificity); several indexes were proposed to establish the best threshold (Youden, Liu, efficiency). All analyses were performed using the Stata Software (StataCorp, College Station, US). A P<0.05 (two-sided) was considered statistically significant.

## Results

### Study population

Thirty patients with ALI/ARDS[24] were enrolled; 30 consecutive age- and sex-matched patients without ALI/ARDS but under mechanical ventilation were included as controls. Data on the primary outcome were available for all patients. Baseline characteristics and main clinical outcomes are summarized in Table 1.

**Table 1:** Baseline characteristics of the patients. Data are expressed as means±standard deviations, unless otherwise indicated. The body-mass index is the weight in kilograms divided by the square of the height in meters. Lung injury (or Murray) score can range from 0 to 4, with higher values indicating more severe injury. Percentages may not exactly total 100% because of rounding.

Characteristic	ARDS group	CONTROL group	P
N	30	30	
Male sex, n (%)	18 (60)	15 (50)	0.4
Age (years)	63±13	59±16	0.3
Body mass index (kg/m^2^)	25.5±7	25.1±4	0.8
Sequential Organ Failure Assessment score	10.5±3.6	9±3.9	0.1
Acute Physiology and Chronic Health Evaluation II score	23.9±9	22.8±7	0.6
Mean arterial pressure (mmHg)	76±7	76±9	0.
Lung injury score	2.9±0.8	1.5±0.9	<0.0001
**Coexisting conditions, n (%)**			
- Atherosclerosis	7 (23)	7 (23)	1
- Hypertension	12 (40)	12 (40)	1
- Current smoking	5 (17)	5 (17)	2
- COPD	5 (17)	5 (17)	1
- Asthma	1 (3)	1 (3)	1
- Lung fibrosis	0 (0)	1 (3)	0.3
- Hematologic neoplasms	6 (20)	7 (23)	0.8
- Dyslipidemia	8 (27)	4 (14)	0.2
- Chronic renal failure	1 (3)	0 (0)	0.3
- Diabetes	6 (20)	5 (17)	0.8
**Associated conditions, n (%)**			
- Sepsis	28 (93)	22 (76)	0.02
- Severe sepsis	25 (83)	17 (57)	0.6
- Septic shock	18 (60)	14 (47)	0.3
- SIRS	29 (97)	22(73)	0.01
**Cause of ICU admission, n (%)**			
- Pneumonia	19 (63)	11 (37)	0.0
- Aspiration	1 (3)	2 (7)	1
- Severe trauma	1(3)	0 (0)	1
- Non-pulmonary sepsis	7 (23)	13 (43)	0.1
- Pancreatitis	0	3 (10)	0.2
- Postoperative of major surgery	0	7 (23)	0.01
- Non traumatic coma	1 (3)	4 (13)	0.2
**Ongoing therapy, n (%)**			
- Statin	4 (13)	4 (13)	1
- Cisatracurium	22 (73)	3 (10)	<0.00
- Corticosteroid	12 (40)	3 (10)	0.007
- Enteral tube feeding	22 (73)	24 (80)	0.5
**Respiratory parameters**			
- PaO_2_/FiO_2_ ratio	116±42	267±72	<0.000001
- Tidal volume (ml/kg ideal body weight)	6.8±1.0	7.4±1.2	0.1
- Inspiratory plateau pressure (cmH_2_O)	27±4	23±5	0.0001
- Positive end-expiratory pressure (cmH_2_O)	13±4	9±	0.00009
**Clinical outcomes at day 28**			
- Ventilator-free days	7±9	7±9	0.9
- Survivors, n (%)	18 (60)	22 (73)	0.3

Nine ARDS patients (30%) had baseline PaO_2_/FiO_2_ between 200 and 300, and 23 patients (76%) were diagnosed with nonfocal lung morphology. First samples for the study were drawn a mean of 33 hours after intubation (SD 39), with no difference between groups (P = 0.7).

### Levels of sRAGE

**Fig 1:**
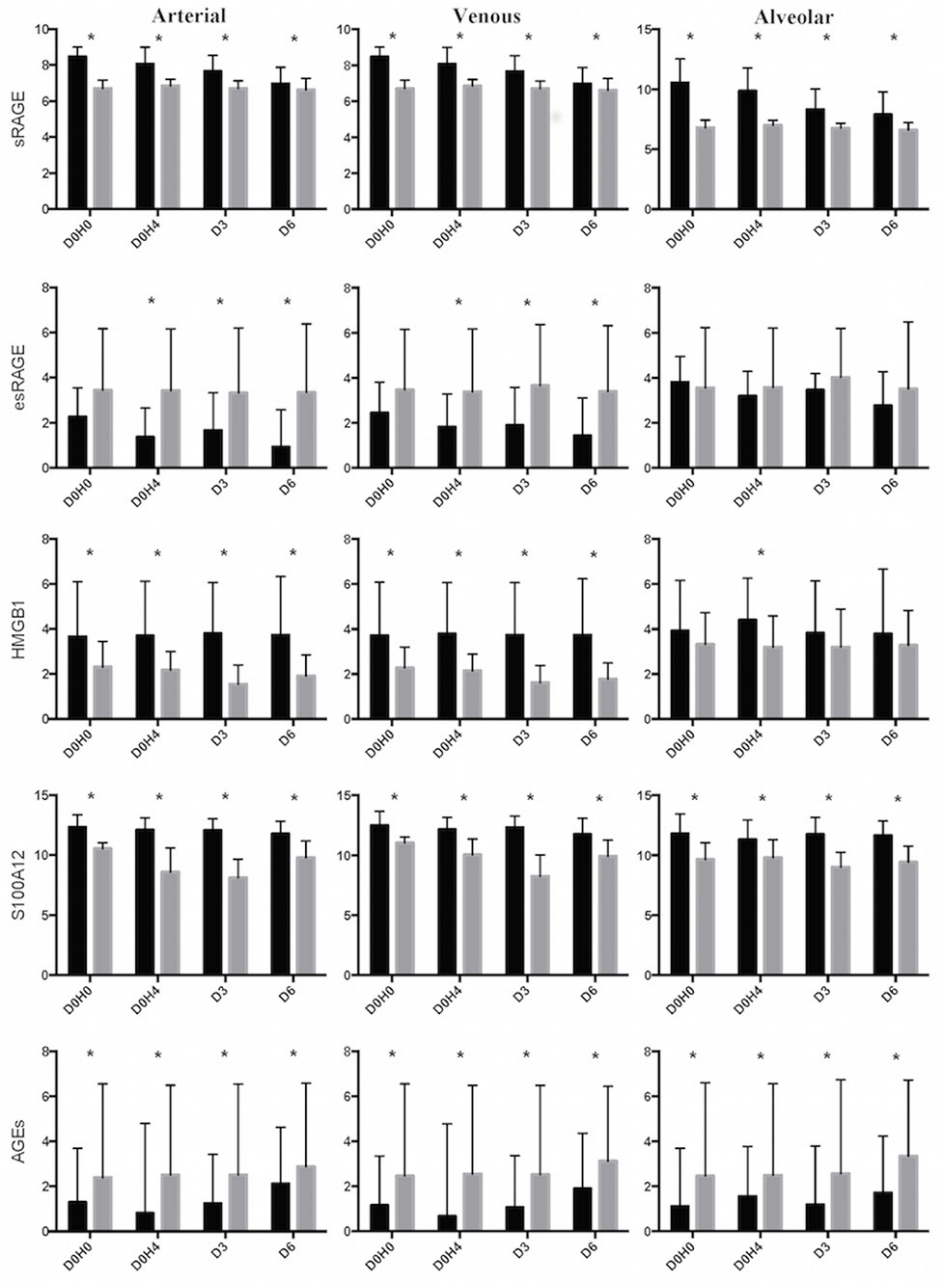
Log-transformed arterial, central venous and alveolar levels of sRAGE (in pg/ml), esRAGE (in ng/ml), HMGB1 (in ng/ml), S100A12 (in pg/ml) and AGEs (in μg/ml) in patients with ARDS (black bars) and controls over time (grey bars). D0H0: inclusion; D0H4: 4 hours after inclusion; D3: day 3; D6: day 6). Values are reported as means±standard deviations. *P<0.05 between groups).

Arterial, central venous and alveolar levels of sRAGE were higher in ARDS patients than in controls at all timepoints (Fig 1 and S1 Table). When samples from all timepoints were analyzed together, venous-to-arterial difference in sRAGE levels was higher in controls (56 [4–210] pg/ml) than in patients with ARDS (-52 [–661–358] pg/ml, P<0.001). The alveolar-to-arterial ratio of sRAGE levels was higher in ARDS patients than in control at baseline (9.2 [2.4–49.6] vs. 0.9 [0.8–2.3], respectively, P<0.001), on D0H4 (7.4 [2.1–25.2] vs. 1.1 [0.9–4.2], P<0.001), D3 (2.6 [0.5–6.4] vs. 0.6 [0.3–1.1], P<0.001) and D6 (2.5 [0.8–6.7] vs. 1.6 [0.03–1.6], P<0.0001). In our population, there was no difference in baseline arterial sRAGE levels between septic (n = 51) and non-septic (n = 9) patients (2508 [978–6234] vs. 1096 [702–1815] pg/ml, respectively, P = 0.09). As well, there was no difference in central venous (P = 0.1) and alveolar levels of sRAGE between groups at baseline (P = 0.1 for both). There was no difference in baseline arterial sRAGE between patients receiving and those not receiving corticosteroids at randomization (2820 [1492–6278] versus 1096 [793–4654] pg/ml, respectively, P = 0.07); no difference was found as well in central venous (P = 0.06) and alveolar (P = 0.2) sRAGE between groups at baseline.

### Levels of esRAGE

Arterial and venous levels of esRAGE were lower in ARDS patients than in controls at all timepoints, but this difference did not reach significance for alveolar esRAGE (Fig 1 and S1 Table). Alveolar esRAGE levels were overall lower in ARDS patients than in controls, except at baseline. When samples from all timepoints were analyzed together, venous-to-arterial difference in esRAGE levels was higher in the ARDS group than in controls (0.3 [-0.04–5.5] vs. 0.06 [-0.08–0.2] ng/ml, P = 0.006). In ARDS patients, arterial sRAGE/esRAGE ratio was higher than in controls at baseline (425 [136–1414] vs. 8 [3–594] pg/ml, P<0.001), on D0H4 (614 [183–1778] vs. 10 [3–543] pg/ml, P<0.001), D3 (336 [106–1366] vs. 6 [3–679] pg/ml, P<0.001) and D6 (458 [149–1269] vs. 6 [3–621] pg/ml, P<0.001). The alveolar-to-arterial ratio of esRAGE levels was higher in ARDS patients than in controls at baseline (3.5 [1.5–12.2] vs. 1 [0.99–1], respectively, P<0.0001), on D0H4 (4.9 [2.3–15] vs. 1 [1–1], P<0.0001), D3 (25.5 [1.9–37.4] vs. 1 [1–2], P = 0.009) and D6 (5.9 [1.1–18.5] vs. 1 [0.3–1], P<0.0001).

### Levels of HMGB1

Plasma levels of HMGB1 were significantly higher in ARDS patients than in controls at all timepoints (Fig 1 and S1 Table), but alveolar HMGB1 was higher in patients with ARDS than in controls only on D0H4. When samples from all timepoints were analyzed together, venous-to-arterial difference in HMGB1 levels was lower in ARDS patients than in controls (-0.1 [-0.6–0.08] vs. 0.09 [-0.9–1.7] ng/ml, P = 0.01). The alveolar-to-arterial ratio of HMGB1 was lower in ARDS patients than in control at baseline (1 [1–1.3] vs. 1.9 [0.9–10.5], respectively, P = 0.01), on D3 (0.9 [0.9–1] vs. 2.9 [1.4–20.9], P<0.0001) D6 (1 [0.3–1] vs. 3.1 [1.1–16.4], P<0.0001), but not on D0H4 (1.1 [1–1.6] vs. 1.5 [0.9–14.4], P = 0.6).

### Levels of S100A12

Levels of S100A12 were higher in ARDS patients than in controls at all timepoints (Fig 1 and S1 Table). When samples from all timepoints were pooled, venous-to-arterial difference in S100A12 levels was similar in the ARDS group and in controls (15334 [–3500–60500] vs. 6059 [1017–20460] pg/ml, P = 0.06). The alveolar-to-arterial ratio of S100A12 was higher in ARDS patients than in controls at baseline (1 [0.3–1.8] vs. 0.2 [0.2–1], respectively, P = 0.03) and on D6 (1.1 [0.4–1.8] vs. 0.8 [0.02–1.6], P = 0.04), but it was lower in ARDS patients than in controls on D0H4 (1 [0.2–1.5] vs. 3.4 [0.8–8.2], P<0.0001), D3 (0.9 [0.7–2.1] vs. 1.5 [0.9–5.7], P = 0.01).

### Levels of AGEs

Levels of AGEs were lower in ARDS patients than in controls at all timepoints (Fig 1 and S1 Table). When samples from all timepoints were analyzed together, venous-to-arterial difference in AGEs levels was lower in the ARDS group than in the control group (0.01 [-0.02–0.05] vs. 0.03 [0.02–0.08] μg/ml, P = 0.002). The alveolar-to-arterial ratio of AGEs levels was not significantly different between ARDS patients and controls at baseline (1.1 [0.6–1.9] vs. 1 [1–1], respectively, P = 0.6), on D0H4 (1 [0.5–1.8] vs. 1 [1–1], P = 0.9), D3 (1 [0.6–2.2] vs. 1 [0.9–1], P = 0.9) or on D6 (1 [0.7–1.9] vs. 1 [0.2–1], P = 0.3).

### Correlation of biological markers with the diagnosis of ARDS, lung injury morphology, severity and outcome

**Fig 2:**
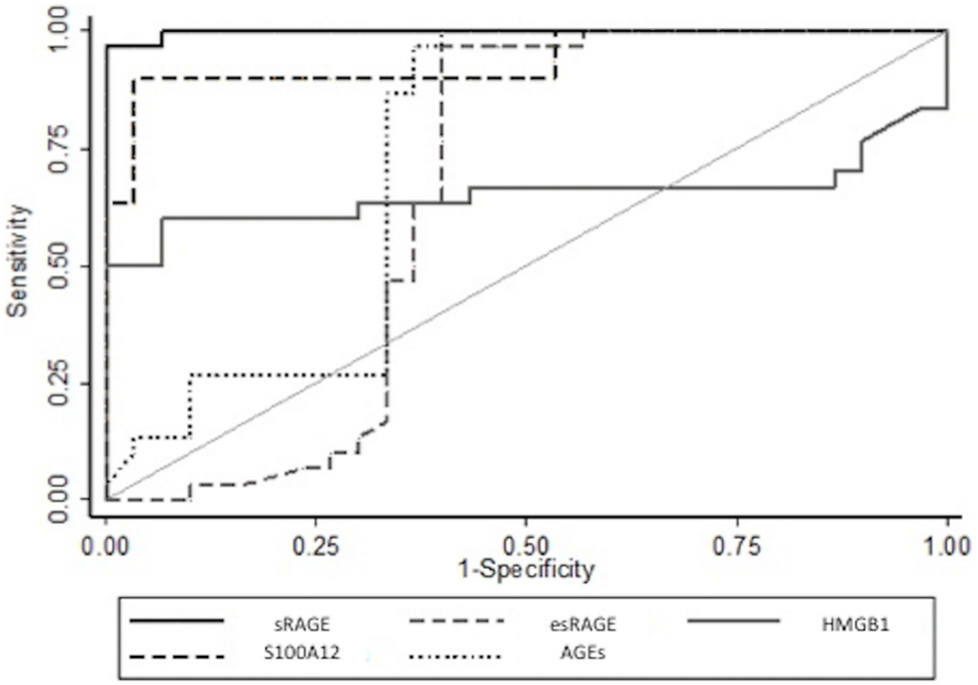
Receiver-operating characteristic curves of baseline arterial levels of sRAGE, esRAGE, HMGB1, S100A12 and esRAGE in differentiating between the presence and absence of ARDS on day 0.

The AUC when baseline arterial sRAGE was used to differentiate the presence from the absence of ARDS was 0.99 (95% CI, 0.99–1). The ability of baseline arterial levels of other markers to discriminate between patients with or without ARDS was also determined; AUC were 0.94 (95% CI, 0.87–1), 0.73 (95% CI, 0.59–0.88), 0.65 (95% CI, 0.49–0.81), 0.65 (95% CI, 0.49–0.81) for baseline arterial S100A12, AGEs, HMGB1 and esRAGE, respectively (Fig 2).

**Fig 3:**
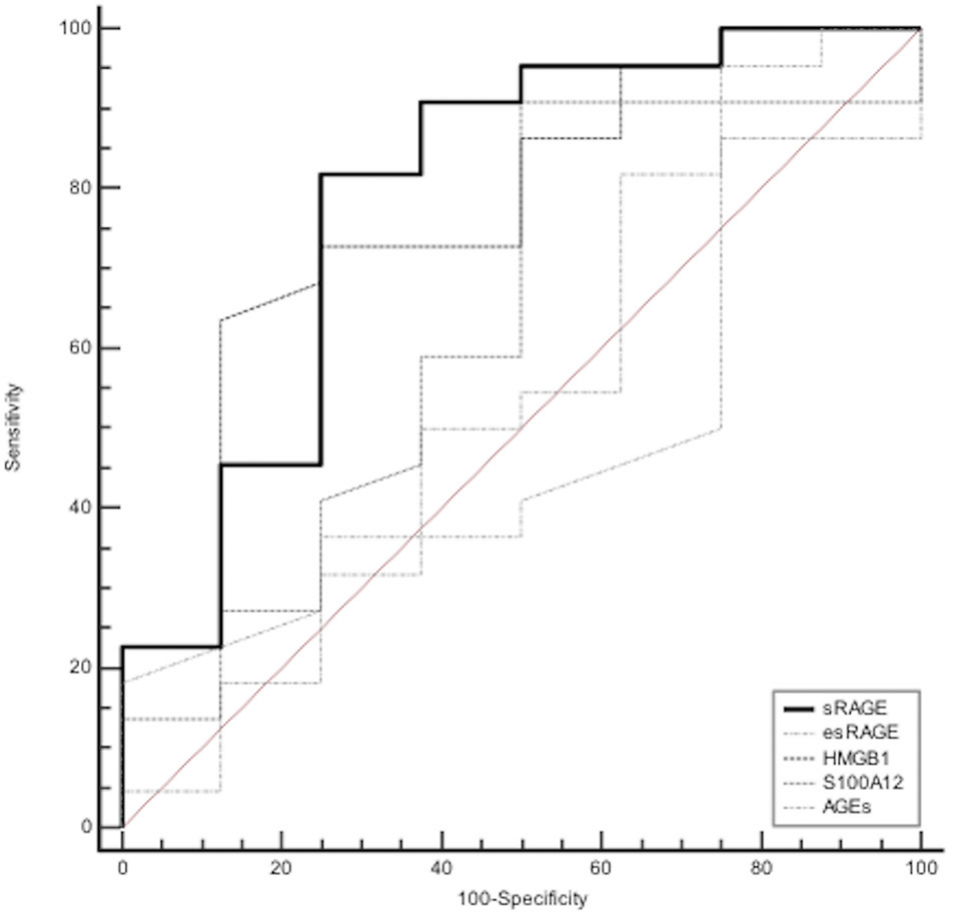
Receiver-operating characteristic curves of baseline arterial levels of sRAGE, esRAGE, HMGB1, S100A12 and esRAGE in differentiating between the presence and absence of nonfocal loss-of-aeration in computed tomography scan morphology studies on day 0 in patients with acute respiratory distress syndrome.

**Fig 4:**
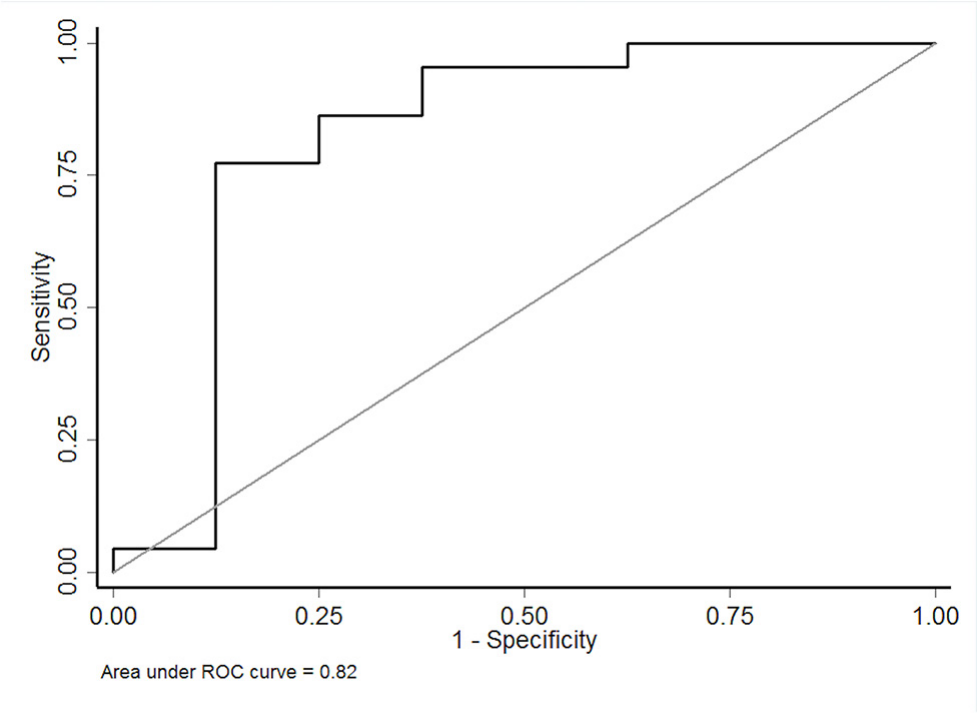
Receiver-operating characteristic curves of the combination of baseline arterial sRAGE, esRAGE, HMGB1, S100A12 and esRAGE in differentiating between the presence and absence of nonfocal loss-of-aeration in computed tomography scan morphology studies on day 0 in patients with acute respiratory distress syndrome.

The AUC when baseline arterial sRAGE was used to differentiate the presence from the absence of nonfocal ARDS was 0.79 (95% CI, 0.6–0.92); a cut-off value of 3494 pg/mL had a sensitivity of 82% (95% CI, 60–95) and a specificity of 75% (95% CI, 35–97). The ability of baseline arterial levels of esRAGE, HMGB1, S100A12 and AGEs to discriminate between nonfocal and focal ARDS was also determined; AUC were 0.65 (95% CI, 0.39–0.91), 0.63 (95% CI, 0.38–0.88), 0.49 (95% CI, 0.24–0.74), 0.48 (95% CI, 0.23–0.74) for baseline arterial HMGB1, S100A12, AGEs and esRAGE, respectively (Fig 3). Next, baseline arterial levels of all markers were combined using logistic regression (multivariate model). The ability of this combination to discriminate between nonfocal and focal ARDS was determined, with an AUC of 0.82 (95% CI, 0.59–1)(Fig 4).

Arterial levels of sRAGE were associated with ARDS severity, as assessed by PaO_2_/FiO_2_ ratio (Spearman’s ρ = -0.36; 95% CI -0.54 to -0.16; P = 0.0008) and lung injury score (ρ = 0.36; 95% CI 0.11 to 0.57; P = 0.006). Baseline arterial S100A12 levels also correlated with PaO_2_/FiO_2_ (ρ = -0.47; 95% CI -0.71 to -0.13; P = 0.008) in ARDS patients. Despite multivariate analysis found colinearity between the 5 markers measured in arterial blood from ARDS patients at baseline, sRAGE, esRAGE, S100A12 and AGEs were associated with PaO_2_/FiO_2_ (P = 0.006, 0.012, 0.002 and 0.006, respectively), but HMGB1 was not (P = 0.3).

No correlation was found significant between lung injury score and esRAGE, HMGB1, S100A12 or AGEs levels. Baseline sRAGE/esRAGE ratio was higher in the ARDS group than in controls (425 [136–1414] vs. 8 [3–594] pg/ml, P<0.001), but was not associated with lung injury severity, number of ventilator-free days or 28-day survival. Baseline arterial sRAGE, esRAGE, HMGB1, S100A12 or AGEs were similar between survivors and non-survivors on day 28 in ARDS patients (P = 0.2, 0.09, 0.3, 0.9 and 0.2, respectively).

## Discussion

We report RAGE isoforms and main ligands levels in the arterial blood, the superior vena cava circulation and the alveolar fluid from ARDS patients, as compared with mechanically ventilated controls, along with their kinetics and association with severity/outcome.

Overall, ARDS patients had higher sRAGE, HMGB1 and S100A12 levels than controls. Inversely, esRAGE and AGEs levels were lower in ARDS patients. A phenotype comprising elevated sRAGE, HMGB1, S100A12 and decreased esRAGE, AGEs could therefore help to characterize patients with or without ARDS. Moreover, increased sRAGE, HMGB1 and S100A12 levels were correlated with nonfocal ARDS and severity. Such findings reinforce previous data suggesting a role for RAGE/HMGB1/S100A12 axis in ARDS[30, 31], and a role for sRAGE as a marker of lung damage[12]. Nevertheless, more work is still needed for us to better understand the implications of RAGE axis in the pathophysiology of alveolar injury and repair, and to facilitate the design of future trials of new ARDS biomarkers or treatments.

Interestingly, blood sRAGE/esRAGE ratio was higher in ARDS patients than in controls at all timepoints. Even though distinct mechanisms are responsible for their release, both isoforms may act as decoys that prevent RAGE interaction with its ligands, thus playing a critical role in RAGE-ligand axis modulation[32]. Levels of sRAGE/esRAGE could represent innate markers of vulnerability to disease or its severity[33], but further research is warranted to explore sRAGE- or esRAGE-mediated mechanisms leading to alveolar inflammation[34]. Our results also support an alveolar source for sRAGE and esRAGE during ARDS, as suggested by higher alveolar-to-plasma ratios for RAGE isoforms than for RAGE ligands in ARDS patients. ARDS patients had higher venous-to-arterial differences in esRAGE levels, but venous-to-arterial differences in sRAGE, HMGB1 and AGEs levels were lower than controls. Even though our study was not specifically designed to address this issue in ARDS, lower venous-to-arterial differences could suggest increased synthesis of sRAGE, HMGB1 and AGEs in the alveolar compartment, and higher differences are compatible with increased accumulation (and/or decreased production) of alveolar esRAGE.

Our study has limitations. First, study design is descriptive and we did not explore how RAGE isoforms and main ligands are released and regulated during ARDS; nevertheless, this first report of the kinetics of the main actors of RAGE axis in multiple compartments should facilitate and stimulate future studies. Second, statistical power has been rigorously calculated *a priori* to detect differences in baseline arterial sRAGE levels between groups. As sRAGE has already been successfully assessed as a diagnostic marker for ARDS[10, 12], we hypothesize that differences in other markers should be considered as relevant, despite not being supported by rigorous power calculation for each of them taken individually. Also, most ARDS patients from our cohort were admitted with a diagnosis of sepsis or pneumonia, yet septic status seems unlikely to influence plasma sRAGE[12]. In addition, we found no difference in baseline arterial, alveolar or central venous levels of sRAGE between septic and non-septic patients, as well as between patients receiving or not receiving corticosteroids at baseline. In our study, patients were diagnosed with ALI/ARDS based on 1994 definition[24]; nevertheless, our results could be extrapolated to patients diagnosed with ARDS based on Berlin definition[3], as all of them had a PEEP>5 cmH_2_O, mean PaO_2_/FiO_2_ ratios compatible with moderate ARDS, and the identification of a risk factor in previous 7 days[3]. As numerous conditions may influence RAGE expression[13, 21], the study was designed to exclude patients with such conditions. Finally, we found no correlation between baseline levels of RAGE isoforms or ligands and main clinical outcomes; however, such outcomes were not assessed here as primary endpoints. Still, prognostic values of RAGE axis mediators cannot be ruled out and should be further assessed with appropriate methodology.

In conclusion, we report the first kinetics study of arterial, central venous and alveolar levels of RAGE main isoforms and ligands during ARDS. A biological phenotype comprising elevated sRAGE, HMGB1 and S100A12 along with decreased esRAGE and AGEs was found to distinguish patients with ARDS from those without. Baseline arterial levels of sRAGE, esRAGE S100A12 and AGEs are correlated with lung injury severity as assessed by PaO_2_/FiO_2_ ratio; baseline sRAGE, HMGB1 and S100A12 may also help to better discriminate nonfocal from focal ARDS, as assessed by CT-scan. Therefore, our findings support a major role for the RAGE/HMGB1/S100A12 axis in lung injury and should prompt future studies aimed at further elucidating this role during ARDS.

## Supporting Information

S1 Checklist STROBE Checklist for cohort studies. (DOC)

S1 Protocol Study Protocol (english version, protocol amendment, ethics committee approval) (DOC)

S1 Table Levels of sRAGE, esRAGE, HMGB1, S100A12 and AGEs (median [Interquartile]) at various study timepoints in patients with or without acute respiratory distress syndrome. (DOCX)

## References

[1] Brun-Buisson C, Minelli C, Bertolini G, Brazzi L, Pimentel J, Lewandowski K, et al Epidemiology and outcome of acute lung injury in European intensive care units. Results from the ALIVE study. Intensive Care Med. 2004;30(1):51–61. 10.1007/s00134-003-2022-6 . 14569423

[2] Rubenfeld GD, Caldwell E, Peabody E, Weaver J, Martin DP, Neff M, et al Incidence and outcomes of acute lung injury. N Engl J Med. 2005;353(16):1685–93. 10.1056/NEJMoa050333 . 16236739

[3] Ranieri VM, Rubenfeld GD, Thompson BT, Ferguson ND, Caldwell E, Fan E, et al Acute respiratory distress syndrome: the Berlin Definition. JAMA. 2012;307(23):2526–33. 10.1001/jama.2012.5669 . 22797452

[4] Ware LB, Matthay MA. The acute respiratory distress syndrome. N Engl J Med. 2000;342(18):1334–49. 10.1056/NEJM200005043421806 . 10793167

[5] Ventilation with lower tidal volumes as compared with traditional tidal volumes for acute lung injury and the acute respiratory distress syndrome. The Acute Respiratory Distress Syndrome Network. N Engl J Med. 2000;342(18):1301–8. 10.1056/NEJM200005043421801 . 10793162

[6] Papazian L, Forel JM, Gacouin A, Penot-Ragon C, Perrin G, Loundou A, et al Neuromuscular blockers in early acute respiratory distress syndrome. N Engl J Med. 2010;363(12):1107–16. 10.1056/NEJMoa1005372 . 20843245

[7] Guerin C, Reignier J, Richard JC, Beuret P, Gacouin A, Boulain T, et al Prone positioning in severe acute respiratory distress syndrome. N Engl J Med. 2013;368(23):2159–68. 10.1056/NEJMoa1214103 . 23688302

[8] Ware LB, Matthay MA. Alveolar fluid clearance is impaired in the majority of patients with acute lung injury and the acute respiratory distress syndrome. Am J Respir Crit Care Med. 2001;163(6):1376–83. 10.1164/ajrccm.163.6.2004035 . 11371404

[9] Shirasawa M, Naoyuki F, Susumu H, Hideki O, Junko I, Koshi M, et al Receptor for advanced glycation end-products is a marker of type I lung alveolar cells. Genes to cells: devoted to molecular & cellular mechanisms. 2004;9(2):165–74. 15009093 10.1111/j.1356-9597.2004.00712.x

[10] Uchida T, Shirasawa M, Ware LB, Kojima K, Hata Y, Makita K, et al Receptor for advanced glycation end-products is a marker of type I cell injury in acute lung injury. Am J Respir Crit Care Med. 2006;173(9):1008–15. 10.1164/rccm.200509-1477OC 16456142 PMC2662912

[11] Calfee CS, Ware LB, Eisner MD, Parsons PE, Thompson BT, Wickersham N, et al Plasma receptor for advanced glycation end products and clinical outcomes in acute lung injury. Thorax. 2008;63(12):1083–9. 10.1136/thx.2008.095588 18566109 PMC2764528

[12] Jabaudon M, Futier E, Roszyk L, Chalus E, Guerin R, Petit A, et al Soluble form of the receptor for advanced glycation end products is a marker of acute lung injury but not of severe sepsis in critically ill patients. Crit Care Med. 2011;39(3):480–8. 10.1097/CCM.0b013e318206b3ca . 21220996

[13] Schmidt AM, Yan SD, Yan SF, Stern DM. The biology of the receptor for advanced glycation end products and its ligands. Biochimica et Biophysica Acta (BBA)—Molecular Cell Research. 2000;1498(2–3):99–111. 10.1016/s0167-4889(00)00087-2 11108954

[14] Hanford LE, Enghild JJ, Valnickova Z, Petersen SV, Schaefer LM, Schaefer TM, et al Purification and characterization of mouse soluble receptor for advanced glycation end products (sRAGE). J Biol Chem. 2004;279(48):50019–24. 10.1074/jbc.M409782200 15381690 PMC1868562

[15] Johnson MD, Widdicombe JH, Allen L, Barbry P, Dobbs LG. Alveolar epithelial type I cells contain transport proteins and transport sodium, supporting an active role for type I cells in regulation of lung liquid homeostasis. Proc Natl Acad Sci U S A. 2002;99(4):1966–71. 10.1073/pnas.042689399 11842214 PMC122303

[16] Cheng C, Tsuneyama K, Kominami R, Shinohara H, Sakurai S, Yonekura H, et al Expression profiling of endogenous secretory receptor for advanced glycation end products in human organs. Mod Pathol. 2005;18(10):1385–96. 10.1038/modpathol.3800450. . 15933755

[17] Yan SF, Barile GR, D'Agati V, Du Yan S, Ramasamy R, Schmidt AM. The biology of RAGE and its ligands: uncovering mechanisms at the heart of diabetes and its complications. Curr Diab Rep. 2007;7(2):146–53. . 17425919 10.1007/s11892-007-0024-4

[18] Schmidt AM, Yan SD, Yan SF, Stern DM. The multiligand receptor RAGE as a progression factor amplifying immune and inflammatory responses. Journal of Clinical Investigation. 2001;108(7):949–55. 10.1172/jci200114002 11581294 PMC200958

[19] Taguchi A, Blood DC, del Toro G, Canet A, Lee DC, Qu W, et al Blockade of RAGE-amphoterin signalling suppresses tumour growth and metastases. Nature. 2000;405(6784):354–60. 10.1038/35012626 . 10830965

[20] Hofmann MA, Drury S, Fu C, Qu W, Taguchi A, Lu Y, et al RAGE mediates a novel proinflammatory axis: a central cell surface receptor for S100/calgranulin polypeptides. Cell. 1999;97(7):889–901. 10399917 10.1016/s0092-8674(00)80801-6

[21] Bierhaus A, Humpert PM, Morcos M, Wendt T, Chavakis T, Arnold B, et al Understanding RAGE, the receptor for advanced glycation end products. J Mol Med (Berl). 2005;83(11):876–86. 10.1007/s00109-005-0688-7 . 16133426

[22] Geroldi D, Falcone C, Emanuele E. Soluble receptor for advanced glycation end products: from disease marker to potential therapeutic target. Current medicinal chemistry. 2006;13(17):1971–8. . 16842191 10.2174/092986706777585013

[23] Dellinger RP, Levy MM, Carlet JM, Bion J, Parker MM, Jaeschke R, et al Surviving Sepsis Campaign: international guidelines for management of severe sepsis and septic shock: 2008. Crit Care Med. 2008;36(1):296–327. 10.1097/01.CCM.0000298158.12101.41 . 18158437

[24] Bernard GR, Artigas A, Brigham KL, Carlet J, Falke K, Hudson L, et al The American-European Consensus Conference on ARDS. Definitions, mechanisms, relevant outcomes, and clinical trial coordination. American Journal of Respiratory and Critical Care Medicine. 1994;149(3 Pt 1):818–24. 10.1164/ajrccm.149.3.7509706 7509706

[25] Malbouisson LM, Muller JC, Constantin JM, Lu Q, Puybasset L, Rouby JJ, et al Computed tomography assessment of positive end-expiratory pressure-induced alveolar recruitment in patients with acute respiratory distress syndrome. Am J Respir Crit Care Med. 2001;163(6):1444–50. 10.1164/ajrccm.163.6.2005001 . 11371416

[26] Puybasset L, Cluzel P, Gusman P, Grenier P, Preteux F, Rouby JJ. Regional distribution of gas and tissue in acute respiratory distress syndrome. I. Consequences for lung morphology. CT Scan ARDS Study Group. Intensive Care Med. 2000;26(7):857–69. . 10990099 10.1007/s001340051274

[27] Constantin JM, Cayot-Constantin S, Roszyk L, Futier E, Sapin V, Dastugue B, et al Response to recruitment maneuver influences net alveolar fluid clearance in acute respiratory distress syndrome. Anesthesiology. 2007;106(5):944–51. 10.1097/01.anes.0000265153.17062.64 . 17457125

[28] Matthay MA, Wiener-Kronish JP. Intact epithelial barrier function is critical for the resolution of alveolar edema in humans. The American review of respiratory disease. 1990;142(6 Pt 1):1250–7. 10.1164/ajrccm/142.6_Pt_1.1250 2252240

[29] Cohen J. Statistical power analysis for the behavioral sciences 2nd ed. Hillsdale, N.J.: L. Erlbaum Associates; 1988 xxi, 567 p. p.

[30] Pietzsch J, Hoppmann S. Human S100A12: a novel key player in inflammation? Amino Acids. 2009;36(3):381–9. 10.1007/s00726-008-0097-7 . 18443896

[31] Nakamura T, Sato E, Fujiwara N, Kawagoe Y, Maeda S, Yamagishi S. Increased levels of soluble receptor for advanced glycation end products (sRAGE) and high mobility group box 1 (HMGB1) are associated with death in patients with acute respiratory distress syndrome. Clin Biochem. 2011;44(8–9):601–4. 10.1016/j.clinbiochem.2010.12.014 . 21211520

[32] Santilli F, Vazzana N, Bucciarelli LG, Davi G. Soluble forms of RAGE in human diseases: clinical and therapeutical implications. Current medicinal chemistry. 2009;16(8):940–52. . 19275604 10.2174/092986709787581888

[33] Colhoun HM, Betteridge DJ, Durrington P, Hitman G, Neil A, Livingstone S, et al Total soluble and endogenous secretory receptor for advanced glycation end products as predictive biomarkers of coronary heart disease risk in patients with type 2 diabetes: an analysis from the CARDS trial. Diabetes. 2011;60(9):2379–85. 10.2337/db11-0291 21771973 PMC3161327

[34] Schlueter C, Hauke S, Flohr AM, Rogalla P, Bullerdiek J. Tissue-specific expression patterns of the RAGE receptor and its soluble forms—a result of regulated alternative splicing? Biochim Biophys Acta. 2003;1630(1):1–6. . 14580673 10.1016/j.bbaexp.2003.08.008

