# Supplementary material for: Soluble Forms and Ligands of the Receptor for Advanced Glycation End-Products in Patients with Acute Respiratory Distress Syndrome: An Observational Prospective Study
Source: PLoS One. 2015 Aug 14;10(8):e0135857. doi: 10.1371/journal.pone.0135857 (PMC4537285; doi:10.1371/journal.pone.0135857)
Supplement: S1 Protocol — (DOC) [file pone.0135857.s002.doc]

**STUDY PROTOCOL**

**ETUDE DU récepteur des produits de glycation avancée (RAGE) AU COURS DU SYNDROME DE DETRESSE RESPIRATOIRE AIGUE: Evaluation des concentrations plasmatiques et alvéolaires de SES formes solubles et de ses ligands potentiels**

***Soluble Forms and LigaNds of RAGE in ALI/ARDS (SoLIRAGE study)***

Promoteur :

CHU de CLERMONT-FERRAND

Rue Montalembert

BP 69

63003 CLERMONT-FERRAND CEDEX

Investigateur principal :

Monsieur le Docteur JABAUDON Matthieu

Service de Réanimation Adultes et Unité de Soins Continus

Département d’Anesthésie Réanimation

CHU Estaing, CHU CLERMONT-FERRAND

Investigateurs :

Monsieur le Professeur CONSTANTIN Jean-Michel

Service de Réanimation Adultes et Unité de Soins Continus

Département d’Anesthésie Réanimation

CHU Estaing, CHU CLERMONT-FERRAND

Monsieur le Professeur SAPIN Vincent

Laboratoire de Biochimie Médicale et Biologie Moléculaire

CHU Estaing, CHU CLERMONT-FERRAND

Collaborateurs scientifiques :

Madame le Docteur ROSZYK Laurence

Laboratoire de Biochimie Médicale et Biologie Moléculaire

CHU Estaing, CHU CLERMONT-FERRAND

Monsieur le Professeur BAZIN Jean-Etienne Département d’Anesthésie Réanimation

CHU Estaing, CHU CLERMONT-FERRAND

**ENGLISH SHORT VERSION**

**OFFICIAL TITLE**: Soluble Forms and Ligands of the Receptor for Advanced Glycation End Products (RAGE) in the Pulmonary Edema Fluid and Plasma From ICU Patients With ALI/ARDS : an Observational Prospective Study.

**PURPOSE:** RAGE, the receptor for advanced glycation end products, is a novel marker of alveolar epithelial type I cell injury. Soluble RAGE (sRAGE) is elevated in the plasma and in the pulmonary edema fluid from patients with ALI/ARDS, but one should acknowledge that the RAGE/NF-B axis is also involved in the pathophysiology of various other conditions. Few data are available about the levels of soluble forms and ligands of RAGE in the setting of ALI/ARDS. The purpose of this observational prospective study is to describe soluble forms (sRAGE, esRAGE) and ligands of RAGE (HMGB-1, S100A12, AGEs) levels in ICU patients with ALI/ARDS.

**BACKGROUND**: The receptor for advanced glycation end products (RAGE) is now identified as a marker of alveolar type I cell injury. RAGE is a member of the immunoglobulin superfamily that acts as a multiligand receptor and is involved in propagating inflammatory responses. While the precise function of RAGE remains unclear, the elevated levels of RAGE, and its soluble isoform sRAGE, correlate with severity of ALI/ARDS in human and animal studies, and RAGE levels could reflect impaired alveolar fluid clearance. Frequently, the biology of RAGE coincides with settings in which ligands of the receptor accumulate, especially in a proinflammatory environment. More work is needed for us to understand the mechanisms by which RAGE is regulated during ALI/ARDS, especially with regard to the expression of its soluble forms and the involvement of its potential ligands.

**STUDY DESIGN:** This observational prospective clinical study will describe and compare soluble forms (sRAGE, esRAGE) and ligands of RAGE (HMGB-1, S100A12, AGEs) levels in the alveolar edema fluid and in the plasma from ICU patients enrolled within the first 24 hours after onset of ALI/ARDS, and from patients under mechanical ventilation (control group).

Edema fluid and plasma samples will be collected simultaneously on day 1, day 3 and day 6, in order to describe kinetics of evolution of soluble forms and ligands of RAGE levels. Undiluted pulmonary edema fluid samples will be collected in intubated patients only, and blood samples will be simultaneously gathered from indwelling arterial and central venous catheters. The concentrations of soluble forms (sRAGE, esRAGE) and ligands of RAGE (HMGB-1, S100A12, AGEs) will be measured in duplicate by ELISA.

**INCLUSION CRITERIA**

- ICU patients under mechanical ventilation
- Patients within the first 24 hours after onset of ALI/ARDS according to the 1994 American-European Consensus Conference (AECC)

**EXCLUSION CRITERIA**

- Pregnancy
- Acute exacerbation of diabetes
- Dialysis for end-stage kidney disease
- Alzheimer's disease
- Amyloidosis
- Evolutive neoplastic lesion

**SOMMAIRE**

1 RATIONNEL DE L’ETUDE [4](#__RefHeading___Toc135109973)

2 RESUME DE L’ETUDE [7](#__RefHeading___Toc135109974)

3 OBJECTIFS DE L’ETUDE [8](#__RefHeading___Toc135109975)

3.1 Objectif principal [8](#__RefHeading___Toc135109976)

3.2 Objectifs secondaires [8](#__RefHeading___Toc135109977)

4 DESCRIPTION DE L’ETUDE [9](#__RefHeading___Toc135109978)

4.1 Type d’étude [9](#__RefHeading___Toc135109979)

4.2 Lieu de l’étude [9](#__RefHeading___Toc135109980)

4.3 Données à recueillir [10](#__RefHeading___Toc135109981)

4.4 Critères d’évaluation [11](#__RefHeading___Toc135109982)

4.4.1 Critères d’évaluation primaires [11](#__RefHeading___Toc135109983)

4.4.2 Critères d’évaluation secondaires [11](#__RefHeading___Toc135109984)

4.5 Population cible [12](#__RefHeading___Toc135109985)

4.5.1 Critères d’inclusion [12](#__RefHeading___Toc135109986)

4.5.2 Critères de non-inclusion [13](#__RefHeading___Toc135109987)

4.5.3 Critères d’arrêt de participation d’un patient à l’étude [13](#__RefHeading___Toc135109988)

4.6 Durée de l’étude [13](#__RefHeading___Toc135109989)

4.7 Nombre de sujets nécessaires [13](#__RefHeading___Toc135109990)

4.8 Capacité de recrutement du service de réanimation [14](#__RefHeading___Toc135109991)

4.9 Déroulement de l’étude [14](#__RefHeading___Toc135109992)

4.10 Comité de Protection des Personnes (CPP) [18](#__RefHeading___Toc135109993)

5 ANALYSE DE L’ETUDE [18](#__RefHeading___Toc135109994)

5.1 Détermination des concentrations des formes solubles de RAGE (sRAGE et esRAGE) et de ses ligands potentiels (HMGB-1, S100A12, AGEs) [18](#__RefHeading___Toc135109995)

5.2 Analyse statistique des résultats [19](#__RefHeading___Toc135109996)

5.3 Rapport d’analyse [19](#__RefHeading___Toc135109997)

6 RECUEIL DES DONNEES [20](#__RefHeading___Toc135109998)

7 EVALUATION DES SURCOUTS [20](#__RefHeading___Toc135109999)

7.1 Traitements [20](#__RefHeading___Toc135110000)

7.2 Matériel utilisé [20](#__RefHeading___Toc135110001)

7.3 Suivi des patients et prélèvements sanguins [21](#__RefHeading___Toc135110002)

7.4 Dosages et mesures [21](#__RefHeading___Toc135110003)

8 CONSIDERATIONS PRATIQUES [23](#__RefHeading___Toc135110004)

8.1 Déclarations officielles [23](#__RefHeading___Toc135110005)

8.2 Contrat d’assurance [23](#__RefHeading___Toc135110006)

8.3 Consentement éclairé [23](#__RefHeading___Toc135110007)

8.4 Evaluation de la sécurité – Gestion des événements indésirables [24](#__RefHeading___Toc135110008)

8.5 Protection du secret médical [26](#__RefHeading___Toc135110009)

8.6 Contrôle de qualité [27](#__RefHeading___Toc135110010)

8.7 Amendements au protocole [27](#__RefHeading___Toc135110011)

8.8 Divulgation des résultats [27](#__RefHeading___Toc135110012)

8.9 Archivage des documents [28](#__RefHeading___Toc135110013)

9 LISTE DES ANNEXES [28](#__RefHeading___Toc135110014)

# RATIONNEL DE L’ETUDE

Le syndrome de détresse respiratoire aiguë (SDRA ou ARDS, pour *Acute Respiratory Distress Syndrome*) et l’*Acute Lung Injury* (ALI, forme modérée de SDRA) sont des états pathologiques graves associés à des complications sévères comme la défaillance multiviscérale ou le décès. Ce sont de véritables « challenges » en réanimation qui sont caractérisés par le manque d’outils diagnostiques et/ou pronostiques spécifiques.

Le récepteur des produits de glycation avancée (RAGE, pour *Receptor for Advanced Glycation End-products*) est un membre de la superfamille des immunoglobulines qui peut se lier à de multiples ligands (1). La liaison de RAGE avec un de ses ligands s’accompagne d’un signal intracellulaire qui provoque l’activation du facteur de transcription proinflammatoire NF-κB (*nuclear factor-κB*), aboutissant à une activation cellulaire prolongée. Cette activation cellulaire est impliquée dans des processus inflammatoires ou des lésions tissulaires, comme par exemple les lésions microvasculaires du diabète, l’amylose ou la maladie d’Alzheimer. RAGE est faiblement exprimé dans les tissus normaux et au niveau vasculaire ; son expression est stimulée dans les sites où ses ligands s’accumulent (*upregulation*). RAGE reconnaît des structures fibrillaires en feuillets β, comme les produits de glycation avancée (AGEs ou *Advanced Glycation End-products*), les médiateurs pro-inflammatoires « *cytokine-like »* de la famille S100/calgranulin, et la protéine HMGB-1 (high-mobility group B1 ou « *amphoterin* »).

Des publications récentes ont montré que RAGE pouvait être utilisé comme marqueur biologique de la lésion des cellules alvéolaires de type I (2-4). Ainsi, l’augmentation des concentrations alvéolaires et plasmatiques de RAGE semble corrélée à la diminution de la clairance alvéolaire liquidienne au cours du SDRA. Ce marqueur pourrait alors avoir un intérêt diagnostique et/ou pronostique dans cette pathologie.

D’autres études ont montré que RAGE pourrait également présenter un intérêt comme marqueur de sepsis grave et de choc septique. L’activation du facteur nucléaire NF-κB, dépendante de RAGE, joue un rôle central dans un modèle animal de choc septique (5), et semble étroitement corrélée à la mortalité observée. En effet, 80% des rats ayant subi une délétion du gène de RAGE (RAGE -/-) survivaient au choc septique.

Nous avons montré, dans une étude observationnelle pilote en réanimation, dont la promotion était assurée par le CHU de Clermont-Ferrand (6), que l’augmentation des concentrations plasmatiques et alvéolaires de sRAGE (pour *soluble* RAGE) observée au cours du SDRA, n’est pas influencée par l’association à un état septique grave (sepsis grave ou choc septique). En réanimation, sRAGE est élevé chez les patients atteints de SDRA, et cette élévation témoigne plus de l’atteinte épithéliale que de l’implication de RAGE dans les cascades inflammatoires systémiques. Ainsi, sRAGE pourrait être utilisé comme un biomarqueur simple et accessible de l’atteinte de l’épithélium respiratoire alvéolaire, même en cas de sepsis grave associé. De nouvelles études restent néanmoins à réaliser afin de mieux comprendre l’implication de l’axe RAGE- NF-κB dans la pathogénie des lésions alvéolaires au cours du SDRA.

La voie de signalisation centrée sur RAGE est imparfaitement connue à ce jour. RAGE semble avoir des ligands et des voies d’activation variables selon le site d’expression et les mécanismes physiopathologiques des différentes pathologies dans lesquelles il est impliqué (7, 8). Initialement, la liaison de RAGE avec les AGEs et cette voie de signalisation ont été décrites au cours d’études concernant les phénomènes inflammatoires chroniques et les complications macrovasculaires liées au diabète (9, 10). Mais RAGE n’est pas seulement un récepteur des AGEs. RAGE peut également se lier au peptide β-amyloïde (dont l’agrégation caractérise la maladie d’Alzheimer), et au peptide amyloïde A (qui s’accumule au cours de l’amylose systémique). Les protéines S100/Calgranulines, une famille de polypeptides liés au calcium s’accumulant dans le milieu extracellulaire dans les sites d’inflammation, se lient également à RAGE. L’HMGB-1 (*High-Mobility Group Box-1*), ou *amphoterin*, une cytokine pro-inflammatoire liée à l’ADN et libérée par les cellules pendant leur nécrose, est aussi un ligand de RAGE (1). Ainsi, RAGE possède un large répertoire de ligands, ces derniers ayant en commun leur propension à s’accumuler dans les tissus au cours du vieillissement, de pathologies dégénératives chroniques, de l’inflammation et de la réponse de l’hôte. Par conséquent, RAGE doit être considéré comme un récepteur à reconnaissance de motifs, ou *Pattern Recognition Receptor* (PRR), qui présente de nombreuses similarités avec les membres de la famille des *Toll-Like Receptors* (TLRs) (11, 12).

On observe une régulation positive (*upregulation*) de l’expression de RAGE dans les tissus où ses ligands s’accumulent. On peut ainsi imaginer une boucle de rétrocontrôle positif dans laquelle l’interaction du récepteur avec son ligand augmente l’expression du récepteur lui-même, majorant alors l’activation cellulaire induite par RAGE. Le seul mécanisme possible de régulation négative (*downregulation*) de l’expression de RAGE est l’interruption du cycle d’engagement des ligands, par les formes solubles de RAGE (sRAGE) ou par des anticorps anti-RAGE (13, 14).

Certains travaux suggèrent que la plupart des ligands connus de RAGE remplissent également des fonctions intra et extracellulaires indépendamment de RAGE (15, 16). Les ligands de RAGE ont surtout été étudiés à l’échelle de l’ARN messager (ARNm), et peu à l’échelle des protéines. Alors que les protéines S100 ont été localisées au niveau des cellules musculaires lisses, une étude récente révèle que l’expression d’ARNm de ligands de RAGE comme HMGB-1 est détectée de façon importante au niveau de tissus tumoraux pulmonaires (17). Dans la plupart des études, ces ligands de RAGE semblent être « co-localisés » avec RAGE lui-même. Par exemple, la co-localisation de RAGE avec HMGB-1 a été décrite dans les cellules neuronales en cours de développement (18). De même, des études par immunohistochimie retrouvent une co-localisation de RAGE avec les AGEs au niveau des macrophages alvéolaires pulmonaires (19, 20).

L’interaction de RAGE avec ses divers ligands paraît ainsi être directement déterminée par les conséquences physiopathologiques inflammatoires qu’elle engendre. Cela suggèrerait l’existence de mécanismes complexes de régulation de l’activation de RAGE, qui dépendraient du site au niveau duquel se déroule cette activation (1, 21). De plus, de nouvelles études sont nécessaires afin de mieux comprendre les relations entre l’expression de RAGE et de ses principales formes solubles (sRAGE, et esRAGE, pour *endogenous secretory* RAGE), qui peuvent agir comme de véritables leurres, et empêcher l’activation de RAGE, en se liant à ses ligands potentiels, les empêchant alors d’interagir avec RAGE (22). A ce jour, aucune donnée de la littérature scientifique ne permet de savoir avec quel(s) ligand(s) RAGE interagit au niveau pulmonaire au cours du SDRA. Toutefois, même si HMGB-1 semble être un bon candidat, d’autres ligands ont été décrits au niveau pulmonaire, comme les protéines S100A12 (ou EN-RAGE, pour *Extracellular Newly identified RAGE-binding protein*), et les AGEs (dont les principaux sont la pentosidine et la N-ε-Carboxyméthyllysine) (23).

Dans ce contexte, et afin de mieux comprendre RAGE, il nous paraît primordial de décrire, au cours du SDRA, les concentrations plasmatiques et alvéolaires des principales formes solubles de RAGE (sRAGE, esRAGE) et des principaux ligands potentiels de RAGE (HMGB-1, S100A12 et les AGEs).

# RESUME DE L’ETUDE

L’étude observationnelle monocentrique que nous proposons de réaliser a pour but de décrire les concentrations plasmatiques et alvéolaires des formes solubles de RAGE (sRAGE et esRAGE) et des ses principaux ligands potentiels (HMGB-1, S100A12, AGEs,) chez des patients de réanimation atteints de SDRA. Aucune donnée n’est actuellement disponible dans la littérature scientifique sur les ligands de RAGE au niveau pulmonaire et sur les valeurs habituelles des concentrations des isoformes solubles de RAGE et de ses ligands, chez les patients de réanimation atteints de SDRA.

Nous proposons d’effectuer les dosages de ces formes solubles et des ligands de RAGE, par réaction immuno-enzymatique (ou ELISA pour *Enzyme-Linked ImmunoSorbent Assay*), chez des patients de réanimation atteints de SDRA et dans un groupe Contrôle composé de patients de réanimation sous ventilation mécanique, mais sans atteinte respiratoire grave (ALI ou SDRA).

Les mesures des concentrations de sRAGE, esRAGE, HMGB-1, S100A12 et des AGEs seront effectuées en duplicata (ELISA), après prélèvements simultanés de plasma et de liquide alvéolaire (au cours d’une aspiration trachéale effectuée à titre systématique pour un autre motif), dans les 24 heures suivant le diagnostic de SDRA (J0), à J3, et à J6.

Il s’agit d’obtenir les premières données concernant les concentrations plasmatiques et alvéolaires des formes solubles de RAGE, et de ses principaux ligands potentiels, chez des patients atteints de SDRA en réanimation, afin de contribuer à une meilleure compréhension de l’implication de l’axe RAGE- NF-κB au cours de la pathogénie et de la résolution de cette pathologie.

# OBJECTIFS DE L’ETUDE

## Objectif principal

Il consiste à décrire les concentrations plasmatiques des isoformes solubles de RAGE (sRAGE et esRAGE) et de ses ligands potentiels (HMGB-1, S100A12, AGEs) dans deux groupes de patients de réanimation :

- un groupe de patients atteints d’ALI ou de SDRA depuis moins de 24 heures au moment de l’inclusion (24)
- un groupe « Contrôle » de patients de réanimation indemnes d’ALI ou de SDRA, et sous ventilation mécanique depuis moins de 24 heures au moment de l’inclusion.

## Objectifs secondaires

- Une analyse complémentaire sur mesures répétées à J3 et J6 permettra d’évaluer la cinétique d’évolution des concentrations plasmatiques des isoformes solubles de RAGE (sRAGE et esRAGE) et de ses ligands potentiels (HMGB-1, S100A12, AGEs).
- La comparaison des concentrations alvéolaires des isoformes solubles de RAGE (sRAGE et esRAGE) et de ses ligands potentiels (HMGB-1, S100A12, AGEs) à J0 dans les deux groupes, afin de comparer l’intérêt pronostique d’un dosage alvéolaire de ces marqueurs par rapport au dosage plasmatique.
- Préciser l’intérêt d’un gradient alvéolo-plasmatique des concentrations des isoformes solubles de RAGE (sRAGE et esRAGE) et de ses ligands potentiels (HMGB-1, S100A12, AGEs).
- La corrélation entre les concentrations des marqueurs étudiés et l’intensité de l’atteinte respiratoire au cours du SDRA sera évaluée.
- Le caractère prédictif des concentrations plasmatiques et alvéolaires des isoformes solubles de RAGE (sRAGE et esRAGE) et de ses ligands potentiels (HMGB-1, S100A12, AGEs) sur la durée de ventilation mécanique et le taux de mortalité à J28 des patients atteints de SDRA sera recherché. Nous comparerons les taux de mortalité théoriques calculés à partir des scores de gravité disponibles (IGS II, SAPS II, APACHE II) et les taux observés en fonction des concentrations des marqueurs biologiques étudiés.

# DESCRIPTION DE L’ETUDE

## Type d’étude

Il s’agit d’une étude clinique, analytique, observationnelle de cohorte, prospective, monocentrique.

Cette étude entre dans le cadre d’une recherche biomédicale hors produits de santé.

## Lieu de l’étude

Les inclusions des patients se dérouleront dans le service de Réanimation Adultes au CHU de Clermont-Ferrand (CHU Estaing).

Les dosages plasmatiques et alvéolaires des formes solubles de RAGE (sRAGE et esRAGE) et de ses ligands potentiels (HMGB-1, S100A12, AGEs) seront réalisés dans le service du Professeur SAPIN, Laboratoire de Biochimie Médicale et Biologie Moléculaire, CHU de Clermont-Ferrand.

La détermination des concentrations des différents marqueurs étudiés sera effectuée par réaction immuno-enzymatique (ELISA, *Enzyme-Linked ImmunoSorbent Assay*), en utilisant des kits disponibles dans le commerce (ANNEXE VI):

- Human sRAGE Quantikine ELISA Kit, R&D Systems, Minneapolis, MN, USA
- esRAGE ELISA Kit, B-Bridge International, Inc., Mountain View, CA, USA
- HMGB-1 ELISA Kit, IBL International GmbH, Hamburg, Germany
- CircuLex^TM^ S100A12/EN-RAGE ELISA Kit, MBL International Corp., CycLex Co., Ltd., Nagano, Japan
- OxiSelect^TM^ Advanced Glycation End-Products (AGEs) ELISA Kit, Cell Biolabs, Inc., San Diego, CA, USA

## Données à recueillir

- Patients:

Il s’agit de données simples et ne nécessitant aucun prélèvement, dosage ou acte en plus de ceux habituellement effectués chez ce type de patients de réanimation :

- Données du dossier médical : données démographiques, antécédents médico-chirurgicaux, sexe, âge, poids, taille, température corporelle…
- Scores de gravité en réanimation, calculés de façon habituelle chez ces patients : Acute Physiology And Chronic Health Evaluation (score APACHE II), Simplified Acute Physiology Score (score SAPS II), Indice de Gravité Simplifié (IGS II), Lung Injury Score (Score de Murray)
- Paramètres hémodynamiques et ventilatoires
- Bilans biologiques et radiographiques habituellement effectués en réanimation
- Pathologie :

Etiologie suspectée ou avérée de l’état clinique

Traitements associés

- Protocole :

Inclusion des patients dans les 24 heures suivant la survenue de l’ALI ou du SDRA. Les critères diagnostiques utilisés dans l’étude sont ceux décrits par la Conférence de Consensus Américano Européenne de 1994 (24).

Pendant la même période d’inclusion, des patients de réanimation, indemnes d’ALI ou SDRA et sous ventilation mécanique depuis moins de 24 heures, seront successivement inclus dans le groupe Contrôle de l’étude.

A l’inclusion, on pratique simultanément un prélèvement sanguin artériel et un prélèvement de liquide alvéolaire afin de mesurer les concentrations plasmatiques et alvéolaires des formes solubles de RAGE (sRAGE et esRAGE) et de ses ligands potentiels (HMGB-1, S100A12, AGEs). Le prélèvement alvéolaire est effectué au décours d’une aspiration trachéale effectuée de façon systématique pour un autre motif, le plus souvent à visée thérapeutique chez un patient intubé sous ventilation mécanique. Cette aspiration est un acte de soin courant chez les patients de réanimation sous ventilation mécanique.

Les prélèvements plasmatiques seront répétés à J3 et J6 suivant l’inclusion dans l’étude, et les prélèvements alvéolaires seront répétés à J3 et J6, si le patient est toujours intubé.

## Critères d’évaluation

### Critères d’évaluation primaires

Les concentrations plasmatiques à J0 des formes solubles de RAGE (sRAGE et esRAGE) et de ses ligands potentiels (HMGB-1, S100A12, AGEs) dans deux groupes de patients de réanimation :

- un groupe de patients atteints d’ALI ou de SDRA depuis moins de 24 heures au moment de l’inclusion (24)
- un groupe « Contrôle » de patients de réanimation indemnes d’ALI ou de SDRA, et sous ventilation mécanique depuis moins de 24 heures au moment de l’inclusion.

### Critères d’évaluation secondaires

- Une analyse complémentaire sur mesures répétées à J3 et J6 permettra d’évaluer d’éventuelles différences dans la cinétique d’évolution des concentrations plasmatiques des isoformes solubles de RAGE (sRAGE et esRAGE) et de ses ligands potentiels (HMGB-1, S100A12, AGEs).
- La comparaison des concentrations alvéolaires des isoformes solubles de RAGE (sRAGE et esRAGE) et de ses ligands potentiels (HMGB-1, S100A12, AGEs) à J0 dans les deux groupes, afin de comparer l’intérêt pronostique d’un dosage alvéolaire de ces marqueurs par rapport au dosage plasmatique.
- Préciser l’intérêt d’un gradient alvéolo-plasmatique des concentrations des isoformes solubles de RAGE (sRAGE et esRAGE) et de ses ligands potentiels (HMGB-1, S100A12, AGEs).
- La corrélation entre les concentrations des marqueurs étudiés et l’intensité de l’atteinte respiratoire au cours du SDRA sera évaluée.
- Le caractère prédictif des concentrations plasmatiques et alvéolaires des isoformes solubles de RAGE (sRAGE et esRAGE) et de ses ligands potentiels (HMGB-1, S100A12, AGEs) sur la durée de ventilation mécanique et le taux de mortalité à J28 des patients atteints de SDRA sera recherché. Nous comparerons les taux de mortalité théoriques calculés à partir des scores de gravité disponibles (IGS II, SAPS II, APACHE II) et les taux observés en fonction des concentrations des marqueurs biologiques étudiés.

## Population cible

Seuls les patients respectant à la fois les critères d’inclusion et de non-inclusion pourront participer au protocole.

### Critères d’inclusion

- patients du service de Réanimation Adultes du CHU de Clermont-Ferrand (CHU Estaing)

- patients de plus de 18 ans

- patients sous ventilation mécanique

- patients en ALI/SDRA depuis moins de 24 heures (24) (ANNEXE III)

- patients ayant donné leur consentement selon les modalités décrites par la loi de santé publique du 9 août 2004. Si le patient n’est pas conscient au moment de l’inclusion, c’est une personne de confiance (conjoint, enfant ou autre) qui accepte ou non de signer le formulaire de consentement et donc l’inclusion dans l’étude.

- patients bénéficiant d’un régime de Sécurité Sociale

### Critères de non-inclusion

- femme enceinte
- refus du protocole
- âge < 18 ans ou Incapable Majeur
- diabète décompensé
- antécédent connu d’insuffisance rénale chronique traitée par dialyse
- antécédent connu de maladie d’Alzheimer
- antécédent connu d’amylose
- antécédent connu de tumeur néoplasique évolutive

### Critères d’arrêt de participation d’un patient à l’étude

Les critères d’arrêt ou de sortie prématurée de l’étude sont les suivants :

- Retrait du consentement du patient
- Retrait du consentement de la personne de confiance du patient si le celui-ci n’est pas conscient

## Durée de l’étude

La durée totale prévue de l’étude est de 12 mois.

La durée de participation pour chaque patient est de 28 jours, et il n’y a pas de période d’exclusion au-delà de ce délai.

## Nombre de sujets nécessaires

Cette étude permettra d’acquérir les premières données sur les concentrations des formes solubles de RAGE (sRAGE et esRAGE) et de ses ligands potentiels (HMGB-1, S100A12, AGEs) chez des patients de réanimation, et aidera à préciser les déterminants de l’activation de la voie de signalisation impliquant RAGE au cours du SDRA. En particulier, cette étude permettra de dégager le ligand principal, voire les ligands principaux, de RAGE au niveau pulmonaire au cours du SDRA, et de préciser la balance entre les formes solubles de RAGE au cours de cette pathologie pulmonaire grave.

Pour une comparaison simple des concentrations plasmatiques des formes solubles de RAGE (sRAGE et esRAGE) et de ses ligands potentiels (HMGB-1, S100A12, AGEs), une puissance de 90% du test est atteinte avec des groupes de 7 patients chacun.

Les effectifs calculés des groupes sont en réalité sur-échantillonnés en fonction des critères d’évaluation secondaires de l’étude, notamment l’étude des taux de mortalité.

Afin de mettre en évidence une différence statistiquement significative des concentrations plasmatiques à J0 (de 1000 pg/mL) des formes solubles de RAGE (sRAGE et esRAGE) et de ses ligands potentiels (HMGB-1, S100A12, AGEs) entre les 2 groupes, avec une puissance de 90% (risque de deuxième espèce β de 10%), un effectif total de 60 patients est nécessaire, avec :

- 30 patients en ALI ou SDRA
- 30 patients sous ventilation mécanique, sans ALI ou SDRA (groupe Contrôle)

## Capacité de recrutement du service de réanimation

Les admissions de patients dans le service de Réanimation Adultes du CHU de Clermont-Ferrand (CHU Estaing) devraient permettre d’inclure environ 5 à 8 patients atteints d’ALI ou de SDRA par mois, ce qui est compatible avec les objectifs de l’étude.

## Déroulement de l’étude

- Inclusion des patients

L’investigateur vérifie que le patient répond aux critères d’inclusion et de non-inclusion, puis remet au patient une notice d’information (ANNEXE I). Le patient (ou la personne de confiance en cas d’impossibilité de recueil du consentement par le patient) signe le formulaire de consentement en deux exemplaires, dont un lui est remis.

A l’inclusion, on pratique simultanément un prélèvement sanguin artériel et un prélèvement de liquide alvéolaire (à l’occasion d’une aspiration trachéale réalisée pour un autre motif chez des patients intubés et ventilés), afin de mesurer les concentrations plasmatiques et alvéolaires des formes solubles de RAGE (sRAGE et esRAGE) et de ses ligands potentiels (HMGB-1, S100A12, AGEs).

Dans les 2 groupes, les prélèvements plasmatiques et alvéolaires seront répétés au cours du troisième jour (J3) et du sixième jour (J6) suivant l’inclusion dans le protocole afin d’obtenir des renseignements sur la cinétique de l’évolution des concentrations des formes solubles de RAGE (sRAGE et esRAGE) et de ses ligands potentiels (HMGB-1, S100A12, AGEs). A J3 et à J6, les prélèvements alvéolaires ne seront réalisés que chez les patients toujours intubés. En cas d’extubation avant J3 ou J6 chez certains patients inclus dans l’étude, seuls les prélèvements plasmatiques seront alors effectués.

La prise en charge diagnostique et thérapeutique de l’ALI et du SDRA est laissée à la discrétion des praticiens prodiguant les soins aux patients concernés par l’étude. L’application des recommandations consensuelles issues des sociétés savantes pour la prise en charge de l’ALI/SDRA est encouragée.

- Anonymisation des données

Le médecin investigateur donne un code à chaque patient inclus dans l’étude (code composé des initiales du nom et du prénom du patient, suivies des chiffres de son mois et de son année de naissance).

Il est le seul à connaître la correspondance nom du patient – numéro d’anonymat.

Cet anonymat permet de ne pas divulguer l’identité du patient, pour la confidentialité et le respect du secret médical, et de limiter les biais de l’analyse statistique.

- Suivi de l’étude

Les prélèvements suivants sont ceux relevant spécifiquement du protocole. Aucun autre prélèvement particulier ne sera effectué dans le cadre du protocole, en dehors des bilans biologiques réalisés de façon usuelle en réanimation.

- Prélèvements plasmatiques :
  - prélèvement de 2 mL de sang artériel sur tube EDTA (quantité nécessaire par chaque dosage : 50 μL), par l’intermédiaire d’un cathéter artériel déjà en place
  - centrifugation dans les 30 minutes après prélèvement, à 1000 x g pendant 15 minutes, à température ambiante
  - recueil du surnageant et conservation à –80°C au laboratoire de Biochimie, avant dosage.
- Prélèvements alvéolaires :
  - selon une technique validée (11)
  - par un cathéter de 14-French, de longueur 51cm (PharmaPlast, Maersk Medical, Denmark) (utilisé habituellement dans les services de réanimation pour les aspirations trachéales), introduit prudemment à travers la sonde d’intubation jusqu’aux voies aériennes distales
  - application d’une pression négative continue de –400 cm d’H20 pendant 5 à 30 secondes
  - afin de recueillir environ 0,5mL de liquide alvéolaire dans un tube EDTA
  - centrifugation : de 1000 x g pendant 15 minutes, à température ambiante
  - recueil du surnageant et conservation à –80°C au laboratoire de Biochimie, avant dosage.

Au total, pour chaque patient, on procède au maximum aux prélèvements suivants : un prélèvement sanguin (1 tube EDTA de 2 mL) et un prélèvement alvéolaire (1 tube EDTA de 1 mL) à J0, J3 et J6, soit un total de 3x3= 9 mL de collection biologique par patient.

Ces quantités sont nécessaires du fait d’une mesure en double de chaque concentration des formes solubles de RAGE (sRAGE et esRAGE) et de ses ligands potentiels (HMGB-1, S100A12, AGEs), pour chaque prélèvement effectué (*duplicate ELISA*). Cette méthode permet de prendre en compte la variabilité analytique de la méthode de dosage afin d’augmenter la qualité et la performance de la méthode de quantification.

Sur l’étiquette de chaque tube sont notés :

- le code du patient
- le type, la date et l’heure du prélèvement

Les tubes seront immédiatement acheminés par l’investigateur de l’étude au service de Biochimie Médicale et Biologie Moléculaire, CHU Estaing, CHU de CLERMONT-FERRAND, et rapidement centrifugés dans les 15 minutes suivant la réalisation du prélèvement.

Le surnageant sera transféré dans un tube EDTA étiqueté (ANNEXE II) puis placé rapidement à -80°c au laboratoire de Biochimie Médicale et Biologie Moléculaire du CHU de Clermont-Ferrand par le Docteur Matthieu JABAUDON ou le Professeur Vincent SAPIN, ou une autre personne désignée par les 2 précédentes. En effet, les formes solubles de RAGE (sRAGE et esRAGE) et ses ligands potentiels (HMGB-1, S100A12, AGEs) sont des composés labiles, dont la conservation nécessite un sérothèquage efficace, rapide, et à une température de –80°C.

La feuille de prélèvement est conservée puis récupérée en même temps que les tubes par le laboratoire de Biochimie Médicale et Biologie Moléculaire.

- Fin d’étude

La durée de participation à l’étude est de 28 jours par patient. Il n’y a pas de durée d’exclusion après la fin de l’étude.

L’étude pourra être interrompue à tout moment par le médecin investigateur. Dans une telle éventualité, l’investigateur s’efforcera de préciser les motifs de l’abandon de l’étude.

S’agissant d’une étude observationnelle dont le critère de jugement principal n’est pas la mortalité, la participation à d’autres protocoles de recherche clinique est autorisée pour les patients inclus dans l’étude.

- Surveillance et prise en charge thérapeutique des patients

La surveillance et la prise en charge thérapeutique habituelles pour les patients de réanimation seront effectuées par les équipes du service de Réanimation Adultes (CHU Estaing, CHU de Clermont-Ferrand). L’application des recommandations consensuelles issues des sociétés savantes pour la prise en charge de l’ALI et du SDRA et des états septiques graves est encouragée.

## Comité de Protection des Personnes (CPP)

Une demande d’autorisation auprès du Comité de Protection des Personnes « Sud-Est VI » dans le cadre de la recherche biomédicale est en cours pour ce protocole.

# ANALYSE DE L’ETUDE

## Détermination des concentrations des formes solubles de RAGE (sRAGE et esRAGE) et de ses ligands potentiels (HMGB-1, S100A12, AGEs)

La mesure des concentrations plasmatiques et alvéolaires des formes solubles de RAGE (sRAGE et esRAGE) et de ses ligands potentiels (HMGB-1, S100A12, AGEs) sera effectuée au Laboratoire de Biochimie Médicale et Biologie Moléculaire, service du Professeur Vincent SAPIN, CHU Estaing, CHU de Clermont-Ferrand.

Les kits de quantification des différents biomarqueurs étudiés sont ceux disponibles dans le commerce. Leur utilisation sera effectuée selon les instructions de leurs fournisseurs respectifs. (ANNEXE VI)

## Analyse statistique des résultats

La distribution des variables étudiées sera d’abord analysée par un test de Kolmogorov-Smirnov. Le test U de Mann-Whitney sera utilisé pour les comparaisons de variables non paramétriques entre les deux groupes, alors que l’ANOVA sera utilisée pour comparer les données paramétriques. Les proportions entre les groupes seront comparées par un test semi-paramétrique du *χ*2, et le test exact de Fisher sera employé pour les comparaisons de variables catégorielles se divisant en deux types de résultats. Des courbes ROC (*Receiver-operating characteristic*) seront calculées afin de déterminer les valeurs seuils optimales pour les associations de variables ; les valeurs seuils optimales seront sélectionnées grâce à l’étude de l’index de Youden. Afin d’établir des corrélations entre deux variables, le coefficient de corrélation de Pearson sera analysé en cas de variables suivant une distribution normale, et l’analyse du coefficient de corrélation des rangs de Spearman sera effectuée pour les variables ne suivant pas une distribution normale.

L’analyse statistique sera réalisée avec le logiciel MedCalc® (Frank Schoonjans, Mariakerke, Belgium). Une valeur de p inférieure à 0,05 sera considérée comme statistiquement significative.

## Rapport d’analyse

Cette étude fera l’objet de communications écrites ou orales, par le Laboratoire de Biochimie Médicale et Biologie Moléculaire du CHU de Clermont-Ferrand, et par le Département d’Anesthésie Réanimation du CHU Estaing, à Clermont-Ferrand.

# RECUEIL DES DONNEES

La feuille de prélèvement sera complétée avec la date et l’heure de prélèvement. Les autres données analysées dans l’étude seront collectées par le médecin investigateur. Les données seront sauvegardées et stockées en un lieu sécurisé, afin de garantir la confidentialité des données et le respect du secret médical. Les données seront stockées, tout au long de l’étude et au décours, dans un lieu sécurisé (annexe VII).

# EVALUATION DES SURCOUTS

## Traitements

Aucun surcoût lié aux traitements n’est à déclarer.

La prise en charge des patients inclus dans l’étude est la même que la prise en charge habituelle de tout patient hospitalisé dans le service de réanimation.

## Matériel utilisé

Le nombre de tubes Vacutainer® contenant de l’EDTA utilisés pour les prélèvements est supérieur à celui d’un suivi classique. Six prélèvements sont prévus pour chacun des 60 patients à inclure dans l’étude. Ainsi, au total, 360 tubes seront utilisés.

## Suivi des patients et prélèvements sanguins

Il s’agit du suivi habituel pour ce type de patient.

Les prélèvements seront effectués par les IDE, les internes et les médecins des services de réanimation: il y aura un surcroît d’activité à ce niveau (6 prélèvements réalisés dans le cadre de l’étude par patient inclus).

Toutefois, cette surcharge de travail est négligeable dans le cadre d’un fonctionnement habituel d’un service de réanimation.

## Dosages et mesures

Les dosages des formes solubles de RAGE (sRAGE et esRAGE) et de ses ligands potentiels (HMGB-1, S100A12, AGEs) seront réalisés par l’équipe du Professeur Vincent SAPIN, au Laboratoire de Biochimie Médicale et Biologie Moléculaire, CHU Estaing, CHU de CLERMONT-FERRAND.

Chaque dosage sera effectué en double (*duplicate* ELISA), afin de prendre en compte la variabilité analytique de la méthode de quantification.

Ainsi, 12 dosages par marqueur seront effectués au maximum (selon si le patient est toujours intubé à chaque temps de dosage), pour chaque patient des deux groupes, soit 720 dosages au total.

Le prix de revient des différents tests ELISA nécessaires à l’étude est résumé dans le tableau suivant :

| Molécule dosée | Prix de revient par kit de dosage (euro)  (82 dosages effectifs /kit) | Total pour 720 dosages  (9 kits) |
| --- | --- | --- |
| sRAGE | 480 | 4320 |
| esRAGE | 464 | 4176 |
| HMGB-1 | 708 | 6372 |
| S100A12 | 619 | 5571 |
| AGEs | 423 | 3807 |
| **TOTAL** |  | **24246** |

Le caractère labile des marqueurs biologiques étudiés nécessite un sérothèquage efficace, rapide, et à une température de –80°C au laboratoire. Le coût de cette méthode de conservation des prélèvements, prenant en compte le personnel, les consommables et la gestion informatique de la sérothèque, s’élève à 7,50 euros par prélèvement, soit un coût total de 5400 euros pour 720 prélèvements.

Ce projet multidisciplinaire est mené en étroite collaboration avec le Laboratoire de Biochimie Médicale et Biologie Moléculaire du Professeur SAPIN (CHU Estaing, CHU de CLERMONT-FERRAND), et le sérothèquage sera réalisé à titre gracieux dans le cadre de la collaboration entre le Département d’Anesthésie Réanimation du CHU Estaing et le Laboratoire du Professeur SAPIN.

Ainsi, le coût total de l’étude s’élève ainsi à 24 246 euros.

Une demande de financement des coûts liés à l’étude est en cours, dans le cadre d’un Programme Hospitalier de Recherche Clinique auprès de la Délégation Régionale à la Recherche Clinique et à l’Innovation d’Auvergne.

Dans le cas où celle-ci serait refusée, l’étude sera possible par le biais d’autres voies de financement.

Une demande conjointe de co-financement auprès des sociétés savantes nationales (*Société Française d’Anesthésie Réanimation, Société de Réanimation de Langue Française*), européennes (*European Society of Intensive Care Medicine*) et internationales (*American Thoracic Society*) est en cours, afin d’obtenir un budget suffisant pour l’étude, et ainsi proposer de nouvelles investigations sur la thématique du récepteur des produits de glycation avancée (RAGE).

# CONSIDERATIONS PRATIQUES

## Déclarations officielles

Le protocole sera soumis à l’approbation du Comité de Protection des Personnes Sud-Est VI.

En signant ce protocole, le promoteur et les investigateurs s’engagent à réaliser l’étude conformément aux Bonnes Pratiques Cliniques et à la Déclaration d'Helsinki de l’Association Médicale Mondiale (Tokyo 2004, révisée).

Le promoteur s’engage à obtenir une autorisation de l’Agence Française de Sécurité Sanitaire et des Produits de Santé (AFSSAPS) avant le début de l’étude.

## Contrat d’assurance

Conformément aux dispositions de la Loi de Santé publique 2004-806 du 09 août 2004, le promoteur s’engage à contracter une assurance responsabilité civile d’entreprise dans le cadre de la recherche biomédicale (ANNEXE IV).

## Consentement éclairé

L’inclusion d’un patient dans l’étude ne pourra avoir lieu qu’après en avoir informé le patient et avoir reçu son consentement par écrit (notice d’information et formulaire de consentement remis et expliqué par l’investigateur, en ANNEXE I). Toute inclusion effectuée sans l’accord signé du patient est contraire aux dispositions légales en vigueur. Le patient devra recevoir une copie de ce document d’information et de consentement. L’investigateur archivera l’original du document pendant la durée légale de 15 ans, dans les conditions de confidentialité prévues par la loi.

Cependant, dans les situations d’incompétence du patient (dont l’altération des fonctions cognitives ne lui permet pas de consentir), une « personne de confiance » désignée à l’avance par le patient lui-même ou à défaut un membre de sa famille se substituera au patient pour le recueil du consentement. De plus, le caractère d’urgence, la gravité inhérente aux pathologies motivant les admissions en réanimation et l’altération très fréquente de la conscience et/ou du jugement des patients admis en réanimation ne permettent que rarement une information éclairée du patient ou de ses proches. Comme prévu par l’article L.1122-1-2 sur les recherches biomédicales en situation d’urgence, lorsque la situation ne permettra pas le recueil du consentement oral du patient, le protocole sera débuté et le consentement éclairé sera demandé pour la poursuite de l’étude et l’utilisation des données, au patient dès que possible.

## Evaluation de la sécurité – Gestion des événements indésirables

L’investigateur a la responsabilité de rapporter tous les événements indésirables dans le cahier d’observation.

**Définitions :**

**Evénement indésirable** : on définit comme événement indésirable toute manifestation nocive survenant chez une personne qui se prête à une recherche biomédicale, que cette manifestation soit liée ou non à la recherche ou au produit sur lequel porte cette recherche

**Effet indésirable :** l’effet indésirable d’une recherche correspond à tout événement indésirable dû à la recherche.

On classe les effets indésirables graves en sous-classes que voici :

*- Effet indésirable grave* ***attendu*** : lorsqu'il est déjà mentionné dans la version la plus récente de la brochure pour l'investigateur, ou dans le Résumé des Caractéristiques du Produit pour les médicaments ayant une Autorisation de Mise sur le Marché, ou dans la notice d’instruction lorsque la recherche porte sur un dispositif médical qui fait l’objet d’un marquage CE.

*- Effet indésirable grave* ***inattendu*** : si sa nature, sa sévérité ou son évolution ne concorde pas avec les informations relatives aux produits, actes pratiqués et méthodes utilisées au cours de la recherche.

**Evénement ou effet indésirable grave** : tout événement ou effet indésirable qui entraîne la mort, met en danger la vie de la personne qui se prête à la recherche, nécessite une hospitalisation ou la prolongation d’une hospitalisation, provoque une incapacité ou un handicap importants ou durables, ou bien se traduit par une anomalie ou une malformation congénitale.

Les décès, quelle que soit leur cause, y compris lorsqu’ils correspondent à une progression de la maladie traitée, sont considérés comme des évènements graves.

D'autres événements ne répondant pas aux qualifications ci-dessus énumérées, peuvent être considérées comme « *potentiellement graves* », notamment certaines anomalies biologiques. Le jugement médical de l'investigateur ou du promoteur pourra conduire à la déclaration de tels événements de la même façon que les événements « graves ».

**Déclaration des événements indésirables graves :**

**L’investigateur a obligation de déclarer dans les 24h au promoteur tout événement indésirable grave survenu chez tout patient inclus dans une étude**, sur un formulaire “ Evénement indésirable grave”, figurant dans les cahiers d’observation.

L’investigateur doit se prononcer sur la relation de causalité entre l’événement indésirable grave avec la recherche.

Un rapport narratif devra être complété et transmis au promoteur dès l’obtention de nouvelles informations pertinentes. Suivant la nature et la gravité de l’événement, des copies du dossier médical anonymisé du patient peuvent être jointes, ainsi que les résultats des analyses de laboratoire.

A réception du formulaire “ Evénement indésirable grave”, le promoteur analyse cet événement et se prononce sur son imputabilité par rapport à l’étude et sur son caractère inattendu au moyen d’une analyse conjointe avec le Centre Régional de Pharmacovigilance.

Conformément au décret d’application n° 2006-477 relatif aux recherches biomédicales, toutes les **suspicions d’effets indésirables graves inattendus**, feront l’objet d’une **déclaration du Promoteur à l’AFSSAPS, au CPP Sud Est VI**, dès qu’il en a connaissance et au plus tard 7 jours après la survenue de l’événement.

Le promoteur tiendra des registres détaillés de tous les événements indésirables qui lui sont notifiés par le ou les investigateurs.

Une fois par an ou sur demande, le promoteur transmettra à l’AFSSAPS et au CPP Sud Est VI un rapport annuel de sécurité tenant compte de toutes des informations de sécurité disponibles.

Les événements indésirables graves seront suivis tout au long de l’étude. Ils sont ceux habituellement rencontrés chez ce type de patients hospitalisés en service de réanimation, et atteints de pathologies graves mettant souvent en jeu le pronostic vital.

Dans ces conditions, et conformément à l’article R1123-37 alinéa 8, la survenue de ces événements indésirables graves ne donne pas lieu à une déclaration.

## Protection du secret médical

Les données recueillies seront anonymisées. Un code sera en effet attribué à chaque patient.

Seul l’investigateur aura connaissance de la correspondance code – patient, ainsi que les personnes en charge du contrôle de la qualité des dossiers ou les personnes réalisant un audit pour les autorités compétentes. Les données seront stockées, tout au long de l’étude et au décours, dans un lieu sécurisé (annexe VIII).

## Contrôle de qualité

L’investigateur s’engage au strict respect au cours du protocole expérimental, des règles de Bonnes Pratiques Cliniques et de la Loi de Santé publique 2004-806 du 09 août 2004.

L’investigateur se porte garant de l’authenticité des données recueillies dans le cadre de l’étude et accepte les dispositions légales autorisant le promoteur de l’étude à mettre en place un contrôle de la qualité.

## Amendements au protocole

Ils seront proposés d’un commun accord entre le promoteur et l’investigateur et notifiés par écrit. Ils feront, selon la nature de l’amendement, l’objet d’un nouvel avis ou d’une simple notification au CPP, et d’une déclaration d’intention complémentaire à la DGS.

## Divulgation des résultats

Les données seront divulguées par le biais de publications effectuées par le Laboratoire de Biochimie Médicale du CHU de Clermont-Ferrand, et par le service d’Anesthésie Réanimation du CHU Estaing (CHU Clermont-Ferrand).

Les patients seront informés par écrit des résultats de la recherche, à la suite des analyses et de la publication de l’étude.

## Archivage des documents

Les originaux des formulaires de consentement signés, la correspondance et les données sources seront conservés par l’investigateur pendant une durée minimale de 15 ans, de même

que le protocole et ses amendements, toute pièce administrative liée à l’étude, les données recueillies et le rapport d’analyse.

# LISTE DES ANNEXES

ANNEXE I : NOTICE D’INFORMATION AU PATIENT ET FORMULAIRES DE CONSENTEMENT

ANNEXE II : ETIQUETTES

ANNEXE III : CRITERES D’ALI / SDRA

ANNEXE IV : CONTRAT D’ASSURANCE

ANNEXE V : CV DES INVESTIGATEURS

ANNEXE VI : KITS DE DOSAGES QUANTITATIFS UTILISES DANS L’ETUDE

ANNEXE VII : SECURISATION DES DONNEES INFORMATIQUES AU C.H.U. DE CLERMONT-FERRAND

ANNEXE VIII : REFERENCES BIBLIOGRAPHIQUES

**ANNEXE I**

**NOTICE D’INFORMATION AU PATIENT ET FORMULAIRES DE CONSENTEMENT**

**NOTICE D’INFORMATION A LA PERSONNE DE CONFIANCE**

**ETUDE DU récepteur des produits de glycation avancée (RAGE) AU COURS DU SYNDROME DE DETRESSE RESPIRATOIRE AIGUE: Evaluation des concentrations plasmatiques et alvéolaires de SES formes solubles et de ses ligands potentiels**

| **NOTICE D’INFORMATION AU PATIENT** |
| --- |

Le Docteur …………………………. vous a proposé de participer à une recherche organisée par le CHU de Clermont-Ferrand sur l’évaluation du dosage de marqueurs biologiques et vous a précisé que vous étiez libre d’accepter ou de refuser cette proposition.

- Buts de l’étude

Votre état de santé s’accompagne de risques et de complications mettant en jeu le pronostic vital. Le syndrome de détresse respiratoire aigu est une pathologie grave dont les causes sont multiples, et de nombreuses études, qu’elles soient expérimentales ou cliniques, tentent actuellement d’améliorer le diagnostic, la prise en charge et la survie des patients concernés. Nous proposons de mesurer les concentrations de différents marqueurs biologiques, afin de tenter de mieux comprendre les mécanismes impliqués dans la progression de cette pathologie pulmonaire. Cela permettrait de mieux reconnaître type d’atteinte, dans le but d’améliorer à terme leur prise en charge.

- Déroulement de l’étude

Cette étude est réalisée dans les conditions prescrites par la Loi de Santé publique 2004-806 du 09 août 2004, assurant la protection des personnes se prêtant aux recherches biomédicales et a reçu un avis favorable du Comité de Protection des Personnes Sud-Est VI, en date du …../…../………..

La réalisation de cette étude ne modifie en rien le traitement qui vous est nécessaire. Elle entraîne simplement un prélèvement sanguin (par l’intermédiaire d’un cathéter déjà en place) et un prélèvement de liquide alvéolaire (à l’occasion d’une aspiration trachéale réalisée de façon habituelle en réanimation, par exemple en cas d’encombrement bronchique), de quelques millilitres au total, dans les 24 heures suivant la survenue de votre maladie. Ces prélèvements seront répétés aux 3^ème^ et 6^ème^ jours de l’évolution de la maladie. Seul le prélèvement sanguin sera alors réalisé si vous n’êtes plus intubé. Ainsi, et au maximum, 3 prélèvements sanguins et 3 prélèvements de liquide alvéolaires seront réalisés.

La durée totale de l’étude, c’est à dire d’observation et de recueil des données cliniques vous concernant, est de 28 jours au total.

La réalisation de ces prélèvements et votre participation à l’étude ne comportent aucun risque spécifique, en dehors des risques liés à la maladie qui a justifié votre admission en réanimation.

Votre participation volontaire à cette étude peut faire progresser les moyens diagnostiques et thérapeutiques mis à la disposition du Corps Médical, mais vous ne devez en attendre aucun bénéfice direct pour votre état de santé.

- Confidentialité et loi informatique et liberté (CNIL)

Les données scientifiques et médicales vous concernant resteront strictement confidentielles. En accord avec la Loi Informatique et Liberté, le nom des sujets sera systématiquement remplacé par un numéro de code dont la correspondance est connue des seuls investigateurs.

- Décision de participer à l’étude

Vous êtes libre d’accepter ou de refuser de participer à cette étude et votre refus ne modifiera en rien la relation avec votre médecin. Si vous acceptez de participer à cette étude, la réglementation en vigueur réclame votre consentement par écrit. Ce consentement ne constitue en aucune manière une décharge de responsabilité et vous conservez tous vos droits garantis par la loi.

Vous êtes libres de vous retirer de l’étude à tout moment, sans avoir à vous justifier. Ceci ne modifiera en rien votre prise en charge et la relation avec votre médecin.

- Résultats de l’étude

Vous avez le droit d’être informé des résultats globaux de cette étude. Vous pourrez les obtenir, dès qu’ils seront disponibles, auprès du médecin investigateur.

- Coordonnées pour obtenir des informations complémentaires

Votre médecin est à votre disposition pour répondre à toutes vos questions concernant cette étude. Voici ses coordonnées :

Dr JABAUDON : 04 73 75 05 01

Service de Réanimation Adultes et Unité de Soins Continus

CHU Estaing, CHU de Clermont-Ferrand

1 Place Lucie Aubrac

63003 CLERMONT FERRAND Cedex 1

Nous vous remercions pour votre collaboration.

Fait à :……………………

Le :………………………..

Signature du patient ou du représentant

(précédée de la mention « Lu et compris ») :

Signature du patient ou du représentant

(précédée de la mention « Lu et compris ») :

**FORMULAIRE DE CONSENTEMENT**

De *(nom du patient)*:...................................................................................................................

Né(e) le : ……………………………………………………………………………………….

demeurant :…………………………………………………………..…………………………

Le Docteur ................................ m’a proposé de participer à l’étude dénommée « ETUDE DU récepteur des produits de glycation avancée (RAGE) AU COURS DU SYNDROME DE DETRESSE RESPIRATOIRE AIGUE: Evaluation des concentrations plasmatiques et alvéolaires de SES formes solubles et de ses ligands potentiels » organisée par le CHU de Clermont-Ferrand. Le docteur m’a précisé que je suis libre d’accepter ou de refuser, ceci n’affectera en rien nos relations. Les objectifs, les risques, la durée de cette étude et sa réalisation pratique m’ont été clairement expliqués. J’ai bien compris toutes les informations qui m’ont été fournies.

Je pourrai à tout moment demander une information complémentaire au médecin. Je recevrai pendant toute la durée de l’étude et quand je le désirerai, une information sur le déroulement de l’essai. Toute nouvelle information pertinente au cours de l’étude pouvant modifier mon consentement me sera fournie par l’investigateur.

Il m’a été signalé que les données me concernant pourraient faire l’objet d’une information partielle selon les modalités prévues par la loi, sans que mon identité ne soit révélée. J’accepte que ces résultats soient consultés par des personnes soumises au secret professionnel et collaborant à cette recherche et par des personnes habilitées par un médecin pour le compte du promoteur et/ou que ces résultats soient transmis aux Autorités Médicales légalement concernées en France et dans tout autre pays.

J’accepte que les données enregistrées à l’occasion de cette recherche puissent faire l’objet d’un traitement informatisé par le promoteur ou pour son compte. J’ai bien noté que le droit d’accès prévu par la loi « Informatique et Liberté » (CNIL, article 40) s’exerce à tout moment auprès du Docteur ................................................ Je pourrai exercer mon droit de rectification auprès de lui.

Mon consentement ne décharge pas les organisateurs de la recherche de leurs responsabilités. Je conserve tous mes droits garantis par la loi. Si je le désire, je serai libre à tout moment d’arrêter ma participation à cet essai. J’en informerai alors immédiatement l’investigateur.

Je m’engage à ne cacher, aux médecins investigateurs, aucune information relative à mon état de santé et à mes habitudes de vie et à répondre en toute franchise aux questions qui me sont posées.

Je sais que cette étude a reçu l’approbation du CPP (Comité de Protection des Personnes) Sud-Est VI, en date du …../…../………. .

Le CHU de Clermont Ferrand, promoteur de l’étude, a souscrit une assurance responsabilité civile couvrant cette recherche auprès de la SOCIETE HOSPITALIERE D’ASSURANCES MUTUELLES 74 rue Louis Blanc 69456 LYON contrat n° XXXXX.

**J’ACCEPTE DE PARTICIPER À CETTE RECHERCHE DANS LES CONDITIONS PRECISEES DANS LA NOTICE D’INFORMATION QUI M’A ÉTÉ REMISE AVEC CE FORMULAIRE ET QUE J’AI LUE AVEC ATTENTION.**

Fait à :……………………

Le :………………………..

Signature

(précédée de la mention « Lu et compris ») :

L’investigateur :………………………..

Fait à :………………………

Le :…………………………

Signature :

**FORMULAIRE DE CONSENTEMENT DE LA PERSONNE DE CONFIANCE**

Le docteur ……………………..., vous propose,

M/Mme (nom, prénom)………………...………………

en qualité de (lien de parenté avec le patient)………………………………………………., que M/Mme (nom, prénom)………………………………… né(e) le …………………………

demeurant à ……………………………………………………………………………………

participe à l’étude organisée par le CHU de CLERMONT FERRAND dénommée « ETUDE DU récepteur des produits de glycation avancée (RAGE) AU COURS DU SYNDROME DE DETRESSE RESPIRATOIRE AIGUE: Evaluation des concentrations plasmatiques et alvéolaires de SES formes solubles et de ses ligands potentiels ».

En raison de la gravité de son état et de l’urgence médicale, son consentement ne peut pas être recueilli. Conformément à la loi (art. L. L1122-1-2 du Code de la Santé Publique), c’est à vous qu’est demandé l’accord de participation de votre proche à cette recherche.

Le docteur m’a précisé que je suis libre d’accepter ou de refuser ; ceci n’affectera en rien vos relations. Les objectifs, les risques, la durée de cette étude et sa réalisation pratique m’ont été clairement expliqués. J’ai bien compris toutes les informations qui m’ont été fournies.

Je pourrai à tout moment demander une information complémentaire au médecin. Je recevrai pendant toute la durée de l’étude et quand je le désirerai, une information sur le déroulement de l’essai. Toute nouvelle information pertinente au cours de l’étude pouvant modifier mon consentement me sera fournie par l’investigateur.

Il m’a été signalé que les données concernant mon proche pourraient faire l’objet d’une information partielle selon les modalités prévues par la loi, sans que son identité ne soit révélée. J’accepte que ces résultats soient consultés par des personnes soumises au secret professionnel et collaborant à cette recherche et par des personnes habilitées par un médecin pour le compte du promoteur et/ou que ces résultats soient transmis aux Autorités Médicales légalement concernées en France et dans tout autre pays. J’accepte que les données enregistrées à l’occasion de cette recherche puissent faire l’objet d’un traitement informatisé par le promoteur ou pour son compte. J’ai bien noté que le droit d’accès prévu par la loi « Informatique et Liberté » (CNIL, article 40) s’exerce à tout moment auprès du Docteur ................................................Je pourrai exercer mon droit de rectification auprès de lui.

Mon consentement ne décharge pas les organisateurs de la recherche de leurs responsabilités. Mon proche conserve tous ses droits garantis par la loi. J’en informerai alors immédiatement l’investigateur.

Je m’engage à ne cacher, aux médecins investigateurs, aucune information relative à l’état de santé de mon proche et à ses habitudes de vies et à répondre en toute franchise aux questions qui me sont posées.

Je sais que cette étude a reçu l’approbation du CPP (Comité de Protection des Personnes) Sud-Est VI en date du …/…/…….

Le CHU de Clermont Ferrand, promoteur de l’étude, a souscrit une assurance responsabilité civile couvrant cette recherche auprès de la SOCIETE HOSPITALIERE D’ASSURANCES MUTUELLES 74 rue Louis Blanc 69456 LYON contrat n° XXXXX.

J’ACCEPTE QUE MON PROCHE PARTICIPE À CETTE RECHERCHE DANS LES CONDITIONS PRECISÉES DANS LA NOTICE D’INFORMATION QUI M’A ÉTÉ REMISE AVEC CE FORMULAIRE ET QUE J’AI LUE AVEC ATTENTION.

Pour le patient ……………….

Fait à :……………………

Le :………………………..

Signature

(précédée de la mention « Lu et compris ») :

L’investigateur :…………………………..

Fait à :………………………

Le :…………………………

Signature :

**FORMULAIRE DE CONSENTEMENT DU PATIENT**

**pour la poursuite de l’étude et l’utilisation des données**

De : .............................................................................................................................................

Né(e) le : ……………………………………………………………………………………….

demeurant :…………………………………………………………..…………………………

En raison de la gravité de votre état et de l’urgence médicale, votre consentement n’a pas pu être recueilli avant le début de l’étude. Conformément à la loi (art. L. 1122-1-2 du Code de la Santé Publique), nous vous demandons votre accord pour la poursuite de l’étude et l’utilisation des données.

Le Docteur ................................ m’a inclus dans l’étude dénommée « ETUDE DU récepteur des produits de glycation avancée (RAGE) AU COURS DU SYNDROME DE DETRESSE RESPIRATOIRE AIGUE: Evaluation des concentrations plasmatiques et alvéolaires de SES formes solubles et de ses ligands potentiels », organisée par le CHU de Clermont-Ferrand. Le docteur m’a précisé que je suis libre d’accepter ou de refuser de poursuivre l’étude, ceci n’affectera en rien nos relations. Les objectifs, les risques, la durée de cette étude et sa réalisation pratique m’ont été clairement expliqués. J’ai bien compris toutes les informations qui m’ont été fournies.

Je pourrai à tout moment demander une information complémentaire au médecin. Je recevrai pendant toute la durée de l’étude et quand je le désirerai, une information sur le déroulement de l’essai. Toute nouvelle information pertinente au cours de l’étude pouvant modifier mon consentement me sera fournie par l’investigateur.

Il m’a été signalé que les données me concernant pourraient faire l’objet d’une information partielle selon les modalités prévues par la loi, sans que mon identité ne soit révélée. J’accepte que ces résultats soient consultés par des personnes soumises au secret professionnel et collaborant à cette recherche et par des personnes habilitées par un médecin pour le compte du promoteur et/ou que ces résultats soient transmis aux Autorités Médicales légalement concernées en France et dans tout autre pays. J’accepte que les données enregistrées à l’occasion de cette recherche puissent faire l’objet d’un traitement informatisé par le promoteur ou pour son compte. J’ai bien noté que le droit d’accès prévu par la loi « Informatique et Liberté » (CNIL, article 40) s’exerce à tout moment auprès du Docteur ................................................ Je pourrai exercer mon droit de rectification auprès de lui.

Mon consentement ne décharge pas les organisateurs de la recherche de leurs responsabilités. Je conserve tous mes droits garantis par la loi. Si je le désire, je serai libre à tout moment d’arrêter ma participation à cet essai. J’en informerai alors immédiatement l’investigateur. Dans ce cas, les données et les prélèvements biologiques réalisés seront entièrement détruits.

Je m’engage à ne cacher, aux médecins investigateurs, aucune information relative à mon état de santé et à mes habitudes de vies et à répondre en toute franchise aux questions qui me sont posées.

Je sais que cette étude a reçu l’approbation du CPP (Comité de Protection des Personnes) Sud-Est VI en date du …../…../……... .

Le CHU de Clermont Ferrand, promoteur de l’étude, a souscrit une assurance responsabilité civile couvrant cette recherche auprès de la SOCIETE HOSPITALIERE D’ASSURANCES MUTUELLES 74 rue Louis Blanc 69456 LYON contrat n° XXXXXX.

**J’ACCEPTE DE POURSUIVRE MA PARTICIPATION À CETTE RECHERCHE DANS LES CONDITIONS PRECISEES DANS LA NOTICE D’INFORMATION QUI M’A ÉTÉ REMISE AVEC CE FORMULAIRE ET QUE J’AI LUE AVEC ATTENTION. J’ACCEPTE QUE LES DONNEES ME CONCERNANT SOIENT UTILISEES.**

| Fait à :……………………  Le :………………………..  Signature  (précédée de la mention « Lu et compris ») : | L’investigateur :………………………..  Fait à :………………………  Le :…………………………  Signature : |
| --- | --- |

**ETUDE DU récepteur des produits de glycation avancée (RAGE) AU COURS DU SYNDROME DE DETRESSE RESPIRATOIRE AIGUE: Evaluation des concentrations plasmatiques et alvéolaires de SES formes solubles et de ses ligands potentiels**

| **NOTICE D’INFORMATION A LA PERSONNE DE CONFIANCE DU PATIENT** |
| --- |

Le Docteur …………………………. vous a proposé, en tant que personne de confiance de M. ……………….. de donner votre consentement afin que votre proche participe à une recherche organisée par le CHU de Clermont-Ferrand sur l’évaluation du dosage de marqueurs biologiques et vous a précisé que vous étiez libre d’accepter ou de refuser cette proposition.

- Buts de l’étude

L’état de santé de votre proche s’accompagne de risques et de complications mettant en jeu le pronostic vital. Le syndrome de détresse respiratoire aigu est une pathologie grave dont les causes sont multiples, et de nombreuses études, qu’elles soient expérimentales ou cliniques, tentent actuellement d’améliorer le diagnostic, la prise en charge et la survie des patients concernés. Nous proposons de mesurer les concentrations de différents marqueurs biologiques, afin de tenter de mieux comprendre les mécanismes impliqués dans la progression de cette pathologie pulmonaire. Cela permettrait de mieux reconnaître type d’atteinte, dans le but d’améliorer à terme leur prise en charge.

- Déroulement de l’étude

Cette étude est réalisée dans les conditions prescrites par la Loi de Santé publique 2004-806 du 09 août 2004, assurant la protection des personnes se prêtant aux recherches biomédicales et a reçu un avis favorable du Comité de Protection des Personnes Sud-Est VI, en date du ...../…../……….

La réalisation de cette étude ne modifie en rien le traitement qui est nécessaire à votre proche. Elle entraîne simplement un prélèvement sanguin (par l’intermédiaire d’un cathéter déjà en place) et un prélèvement de liquide alvéolaire (à l’occasion d’une aspiration trachéale réalisée de façon habituelle en réanimation, par exemple en cas d’encombrement bronchique), de quelques millilitres au total, dans les 24 heures suivant la survenue de la maladie. Ces prélèvements seront répétés aux 3^ème^ et 6^ème^ jours de l’évolution de la maladie. Seul le prélèvement sanguin sera alors réalisé si votre proche n’est plus intubé. Ainsi, et au maximum, 3 prélèvements sanguins et 3 prélèvements de liquide alvéolaires seront réalisés.

La durée totale de l’étude, c’est à dire d’observation et de recueil des données cliniques concernant votre proche, est de 28 jours au total.

La réalisation de ces prélèvements et la participation de votre proche à l’étude ne comportent aucun risque spécifique, en dehors des risques liés à la maladie qui a justifié son admission en réanimation.

Cette étude peut faire progresser les moyens diagnostiques et thérapeutiques mis à la disposition du Corps Médical, mais vous ne devez en attendre aucun bénéfice direct pour l’état de santé de votre proche.

- Confidentialité et loi informatique et liberté (CNIL)

Les données scientifiques et médicales concernant votre proche resteront strictement confidentielles. En accord avec la Loi Informatique et Liberté, le nom des sujets sera systématiquement remplacé par un numéro de code dont la correspondance est connue des seuls investigateurs.

- Décision de participer à l’étude

Vous êtes libre d’accepter ou de refuser la participation de votre proche à cette étude et ce refus ne modifiera en rien la relation avec le médecin. Si vous acceptez que votre proche participe à cette étude, la réglementation en vigueur réclame votre consentement par écrit. Ce consentement ne constitue en aucune manière une décharge de responsabilité et vous et votre proche conservez tous vos droits garantis par la loi.

- Résultats de l’étude

Votre proche a le droit d’être informé des résultats globaux de cette étude. Il pourra les obtenir, dès qu’ils seront disponibles, auprès du médecin investigateur.

- Coordonnées pour obtenir des informations complémentaires

Un médecin est à votre disposition pour répondre à toutes vos questions concernant cette étude. Voici ses coordonnées :

Dr JABAUDON : 04 73 75 05 01

Service de Réanimation Adultes et Unité de Soins Continus

CHU Estaing, CHU de Clermont-Ferrand

1 Place Lucie Aubrac

63003 CLERMONT FERRAND Cedex 1

Nous vous remercions pour votre collaboration.

Fait à :……………………

Le :………………………..

Signature de la personne de confiance du patient

(précédée de la mention « Lu et compris ») :

**ANNEXE II**

**ETIQUETTES**

**POUR CHAQUE PATIENT :**

| Code patient :  Date :  Heure :  **PRELEVEMENT SANGUIN**  **J0** | Code patient :  Date :  Heure :  **PRELEVEMENT ALVEOLAIRE**  **J0** |
| --- | --- |
| Code patient :  Date :  Heure :  **PRELEVEMENT SANGUIN**  **J3** | Code patient :  Date :  Heure :  **PRELEVEMENT ALVEOLAIRE**  **J3** |
| Code patient :  Date :  Heure :  **PRELEVEMENT SANGUIN**  **J6** | Code patient :  Date :  Heure :  **PRELEVEMENT ALVEOLAIRE**  **J6** |

**ANNEXE III**

**CRITERES de SDRA et d’ALI**

- rapport PaO_2_ /FiO_2_ ≤ 300 (ALI) ou 200 (SDRA),
- infiltrats pulmonaires radiologiques bilatéraux, d’apparition récente,
- absence de défaillance cardiaque gauche clinique, ou PAPO (pression de l’artère pulmonaire occluse) ≤ 18 mmHg, si mesurée.

*selon les critères de la Conférence de Consensus Américano Européenne de 1994 (24)*

**ANNEXE IV**

**CONTRAT D’ASSURANCE**

**ANNEXE V**

**CV DES INVESTIGATEURS**

**CURRICULUM VITAE abrégé des investigateurs**

**Nom** : **JABAUDON Matthieu**

**Fonctions** : Chef de clinique des Universités, Assistant des Hôpitaux en Anesthésie Réanimation (CHU Clermont-Ferrand, Université d’Auvergne Clermont 1)

**N° CNOM**: 63/5679

**Diplômes** :

Maîtrise en science biologiques et médicales (MSBM) : *méthodologie en recherche clinique et épidémiologique*, Université Paris V, Faculté Necker-Enfants Malades (2003)

D.E.S en Anesthésie Réanimation, Université d’Auvergne Clermont 1 (2008)

**Organisme** : CHU Clermont-Ferrand, Université d’Auvergne Clermont 1

**Affiliation** :

INSERM 🞎 CNRS 🞎 Autres (préciser) 🞎

Adresse : Réanimation Adultes et Unité de Soins Continus, Département d’Anesthésie Réanimation, CHU Estaing, 63000 Clermont-Ferrand.

*Téléphone :* 04 73 750 501 *Télécopie*: 04 73 750 500

*Email :* [*mjabaudon@chu-clermontferrand.fr*](mailto:mjabaudon@chu-clermontferrand.fr)

**Publications** :

[1] **Jabaudon M**, Futier E, Roszyk L, Chalus E, Guerin R, Petit A, Mrozek S, Perbet S, Cayot-Constantin S, Chartier C, et al. Receptor for advanced glycation end-products is a biomarker of acute respiratory distress syndrome. **Crit Care Med** 2010; *In Press*.

[2] Constantin JM, Futier E, Cherprenet AL, Chanques G, Guerin R, Cayot-Constantin S, **Jabaudon M**, Perbet S, Chartier C, Jung B, Guelon D, Jaber S, Bazin JE. A recruitment maneuver increases oxygenation after intubation of hypoxemic intensive care unit patients: a randomized controlled study. **Crit Care**. 2010 Apr 28;14(2):R76.

[3] Constantin JM, Futier E, Perbet S, Roszyk L, Lautrette A, Gillart T, Guerin R, **Jabaudon M**, Souweine B, Bazin JE, Sapin V. Plasma neutrophil gelatinase-associated lipocalin is an early marker of acute kidney injury in adult critically ill patients: a prospective study. **J Crit Care**. 2010 Mar;25(1):176.e1-6.

[4] **Jabaudon M**, Bonnin M, Bolandard F, Chanseaume S, Dauphin C, Bazin JE. Takotsubo syndrome during induction of general anaesthesia. **Anaesthesia** 2007;62(9):519-23.

**Date: 08/12/2010 Signature: M JABAUDON**

**CURRICULUM VITAE abrégé des investigateurs**

**Nom** : **CONSTANTIN Jean-Michel**

**Fonctions** : Professeur des Universités / Praticien Hospitalier en Anesthésie Réanimation

**N° CNOM**: 63/4662

**Titres** : Docteur en médecine, Docteur d’Université, Professeur des Universités

**Organisme** : CHU Clermont-Ferrand

**Affiliation** :

INSERM  CNRS  Autres 

Adresse : Réanimation Adultes et Unité de Soins Continus, Département d’Anesthésie Réanimation, CHU Estaing, 63000 Clermont-Ferrand.

*Téléphone :* 04 73 750 501 *Télécopie*: 04 73 750 500

*Email :* [jmconstantin@chu-clermontferrand.fr](mailto:jmconstantin@chu-clermontferrand.fr)

**Principales publications** :

[1] **Constantin JM**, Futier E, Cherprenet AL, Chanques G, Guerin R, Cayot-Constantin S, Jabaudon M, Perbet S, Chartier C, Jung B, Guelon D, Jaber S, Bazin JE. A recruitment maneuver increases oxygenation after intubation of hypoxemic intensive care unit patients: a randomized controlled study. **Crit Care**. 2010 Apr 28;14(2):R76.

# [2] Constantin JM, Jaber S, Futier E, [**Cayot-Constantin S**](http://www.ncbi.nlm.nih.gov.gate2.inist.fr/pubmed?term="Cayot-Constantin S"%5BAuthor%5D), [**Verny-Pic M**](http://www.ncbi.nlm.nih.gov.gate2.inist.fr/pubmed?term="Verny-Pic M"%5BAuthor%5D), Jung B, [**Bailly A**](http://www.ncbi.nlm.nih.gov.gate2.inist.fr/pubmed?term="Bailly A"%5BAuthor%5D), [**Guerin R**](http://www.ncbi.nlm.nih.gov.gate2.inist.fr/pubmed?term="Guerin R"%5BAuthor%5D), [**Bazin JE**](http://www.ncbi.nlm.nih.gov.gate2.inist.fr/pubmed?term="Bazin JE"%5BAuthor%5D). Respiratory effects of different recruitment maneuvers in acute respiratory distress syndrome. Crit Care. 2008;12(2):R50.

[3] **Constantin JM**, Cayot S, Roszyk L, Futier E, Sapin V, Bazin JE, et al. Response to Recruitment Maneuver Influences Net Alveolar Fluid Clearance in acute respiratory distress syndrome. **Anesthesiology**. 2007;106(5):944-51.

[4] Lu Q, **Constantin JM**, Nieszkowska A, Elman M, Vieira S, Rouby JJ. Measurement of alveolar derecruitment in patients with acute lung injury: computerized tomography versus pressure-volume curve. **Crit Care** 2006;10(3):R95.

[5] Malbouisson LM, Puybasset L, **Constantin JM**, Lu Q, Cluzel P, Rouby JJ. Comparison of two CT Scan methods to asses alveolar recruitment in ards. **Am J Respir Crit Care Med** 2001;164(5):2005-12.

**Date: 08/12/2010 Signature: JM CONSTANTIN**

**CURRICULUM VITAE abrégé des investigateurs**

**Nom** : **SAPIN Vincent**

**Fonctions** : ~ Professeur des Universités / Praticien Hospitalier de Biochimie et Biologie

Moléculaire

~ Chef de Service de « Biochimie Médicale et Biologie Moléculaire » du CHU

de Clermont-Ferrand

**Titres** : ~ D.E.S de Biologie Médicale (1996)

~ Thèse d’Université en Biologie Moléculaire et Cellulaire (1999)

~ Habilitation à Diriger les Recherches (2003)

**Organisme** : UMR CNRS 6247, Clermont Université

**Affiliation** :

INSERM 🞎 CNRS X Autres (préciser) 🞎

Adresse : Laboratoire de Biochimie Médicale / Centre de Biologie

CHU Clermont-Ferrand / 63000 Clermont-Ferrand

*Téléphone :* 04 73 75 18 00 *Télécopie*: 04 73 75 18 55

*Email :* [vsapin@chu-clermontferrand.fr](mailto:vsapin@chu-clermontferrand.fr)

**Principales publications** :

[1] MARCEAU G., **SAPIN V.,** JACOMET C., UGHETTO S., CORMERAI L., REGAGNON C., DASTUGUE B., PEIGUE-LAFEUILLE H., BEYTOUT J. , LAUTICHESSE H. (2003) Frequency, risk factors and outcome of hyperlactataemia in HIV-positive persons : implications for the management of treated patients. **Clinical Chemistry**, 49 (7): 1154-1162.

[2] Gallot D., COSTE K., Francannet C., Laurichesse-Delmas H., Boda C., Vanlieferinghen P., Scheye T., Vendittelli F., Déchelotte P., **Sapin V.**, Lémery D. (2005) Antenatal detection and impact on outcome of congenital diaphragmatic hernia : a 12-year experience in Auvergne (France). **European Journal of Obstetrics Gynaecology and Reproductive Biology**: 125(2): 202-205.

[3] **SAPIN V.,** NICOLET L., AUBLET-CUVELIER B., SANGLINE F., ROSZYK L., DASTUGUE B. , GAZUY N., DETEIX P., SOUWEINE B. (2006) Rapid Decrease in plasma D-lactate as an early potential predictor of diminished 28-Day mortality in critically ill spetic shock patients. **Clinical Chemistry and Laboratory Medicine** 44 (4): 492-496*.*

[4] CONSTANTIN JM., CAYOT-CONSTANTIN S., ROSZYK L., FUTIER E., **SAPIN V.,** DASTUGUE B., BAZIN JE., ROUBY JJ. (2007) Response to recruitement maneuver influences net alveolar fluid clearance in acute respiratory distress syndrome. **Anesthesiology** 106: 944-951.

[5] GALLOT D., DE CHAZERON I., BOUSSIRON D., UGHETTO S., VENDITELLI F., LEGROS F., ROSZYK L., LLORCA PM, LEMERY D., **SAPIN V.** (2007) Limits of usual biochemical alcohol markers in cord blood at term: a fetal/maternal population based study. **Clin Chem Lab Med:** 45 (4): 546-548.

**Date: 08/12/2010 Signature: V SAPIN**

**ANNEXE VI**

**KITS DE DOSAGES QUANTITATIFS UTILISES DANS L’ETUDE**

- **Human sRAGE Quantikine ELISA Kit, R&D Systems, Minneapolis, MN, USA**

<http://www.rndsystems.com/pdf/drg00.pdf>

- **esRAGE ELISA Kit, B-Bridge International, Inc., Mountain View, CA, USA**

<http://lifesciences.b-bridge.com/_assets/images/products/K1009-1_manual.pdf>

- **HMGB-1 ELISA Kit, IBL International GmbH, Hamburg, Germany**

<http://www.ibl-international.com/magento/media/catalog/product/S/T/ST51011_IFU_en_HMGB1_ELISA_2010_02.pdf>

- **CircuLex^TM^ S100A12/EN-RAGE ELISA Kit, MBL International Corp., CycLex Co., Ltd., Nagano, Japan**

<http://www.mblintl.com/sites/default/files/datasheets/CY-8058.PDF>

- **OxiSelect^TM^ Advanced Glycation End Product (AGE) ELISA Kit, Cell Biolabs, Inc., San Diego, CA, USA**

http://www.cellbiolabs.com/PDFs/EF4DA364-3048-812A-2EDC45E9F543BF8C.pdf

**ANNEXE VII**

**SECURISATION DES DONNEES INFORMATIQUES AU C.H.U. DE CLERMONT-FERRAND**

| **Sécurisation du réseau au CHU de Clermont-Ferrand** |
| --- |

L’élément technique central de la sécurité est un pare-feu de type Firewall-1 de l’éditeur CheckPoint, leader mondial sur ce marché. Ce firewall est hébergé sur un serveur SecurePlateform, système d’exploitation dont la fiabilité et la sécurité ont été renforcées.

Ce firewall remplit principalement un rôle de filtrage : par défaut, il bloque toutes les connexions qui le traversent, à l’exception de celles qui sont explicitement autorisées.

L’architecture réseau mise en place autour de ce firewall permet de s’assurer que le réseau local du CHU est protégé de la plupart des risques d’intrusion et d’infection par des logiciels malveillants. Une zone démilitarisée (DMZ) a été constituée. Il s’agit d’un réseau tampon dédié à l’hébergement des systèmes devant être accessible depuis internet. Ces systèmes sont ainsi sous le contrôle direct du firewall qui filtre le trafic à destination et en provenance de ces machines.

Concernant l’accès à internet, la sécurité repose sur un serveur proxy Microsoft ISA Server situé sur le réseau local, hors d’atteinte des menaces d’internet. Il permet uniquement de naviguer sur le web, sous condition d’authentification par identifiant et mot de passe personnels. Ainsi, seuls les accès dûment autorisés sont possibles. Sa position de frontal fait que les objets internet qui constitueraient une menace sont interceptés sans jamais atteindre le poste de travail de l’utilisateur. Il joue également un rôle de filtre en interdisant certains sites jugés indésirables.

La messagerie électronique du CHU est protégée par un antivirus eSafe Aladdin dédié. Celui-ci examine systématiquement tous les messages entrants et sortants. Il est capable d’éradiquer un virus contenu dans un message ou de bloquer le message en question. Ce serveur antivirus est situé sur une DMZ dédiée, garantissant que les virus interceptés ne se propagent pas sur d’autres ordinateurs. De plus le serveur de messagerie qui héberge les boîtes aux lettres est situé sur le réseau local et non pas en DMZ, ce qui permet d’éviter tout risque d’interception d’un message depuis l’extérieur.

Les accès distants sont également sous contrôle. Bien sûr, les connexions en provenance d’internet sont concernées et donc soumises au filtrage du firewall. Ceci est également valable pour les accès via numéris ou des réseaux tiers comme le RSS, qui aboutissent en DMZ d’abord, avant de franchir le firewall.


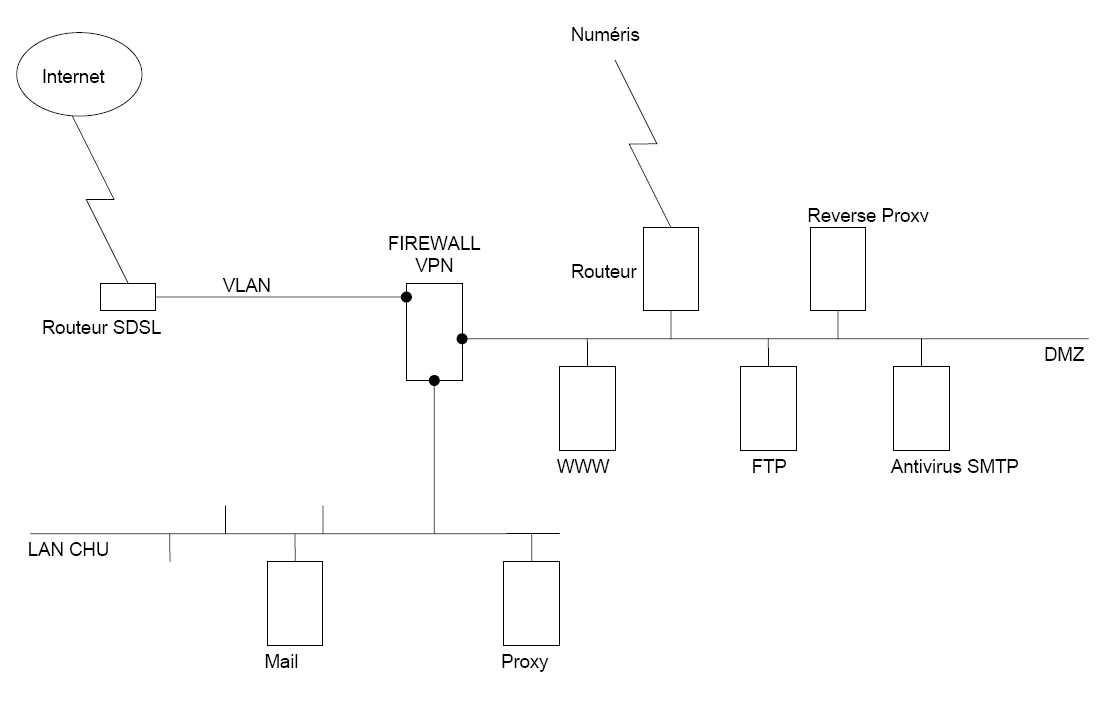


**ANNEXE VIII**

**REFERENCES BIBLIOGRAPHIQUES**

1. Bierhaus A, Humpert PM, Morcos M, Wendt T, Chavakis T, Arnold B, Stern DM, Nawroth PP. Understanding rage, the receptor for advanced glycation end products. *J Mol Med* 2005;83:876-886.

2. Frank JA, Briot R, Lee JW, Ishizaka A, Uchida T, Matthay MA. Physiological and biochemical markers of alveolar epithelial barrier dysfunction in perfused human lungs. *Am J Physiol Lung Cell Mol Physiol* 2007;293:L52-59.

3. Uchida T, Shirasawa M, Ware LB, Kojima K, Hata Y, Makita K, Mednick G, Matthay ZA, Matthay MA. Receptor for advanced glycation end-products is a marker of type i cell injury in acute lung injury. *Am J Respir Crit Care Med* 2006;173:1008-1015.

4. Briot R, Frank JA, Uchida T, Lee JW, Calfee CS, Matthay MA. Elevated levels of the receptor for advanced glycation end products, a marker of alveolar epithelial type i cell injury, predict impaired alveolar fluid clearance in isolated perfused human lungs. *Chest* 2009;135:269-275.

5. Liliensiek B, Weigand MA, Bierhaus A, Nicklas W, Kasper M, Hofer S, Plachky J, Grone HJ, Kurschus FC, Schmidt AM, Yan SD, Martin E, Schleicher E, Stern DM, Hammerling GG, Nawroth PP, Arnold B. Receptor for advanced glycation end products (rage) regulates sepsis but not the adaptive immune response. *J Clin Invest* 2004;113:1641-1650.

6. Jabaudon M, Futier E, Roszyk L, Chalus E, Guerin R, Petit A, Mrozek S, Perbet S, Cayot-Constantin S, Chartier C, Sapin V, Bazin J, Constantin J. Receptor for advanced glycation end-products is a biomarker of acute respiratory distress syndrome. *Crit Care Med* 2010;In Press.

7. Ramasamy R, Yan SF, Schmidt AM. Rage: Therapeutic target and biomarker of the inflammatory response--the evidence mounts. *J Leukoc Biol* 2009;86:505-512.

8. Santilli F, Vazzana N, Bucciarelli LG, Davi G. Soluble forms of rage in human diseases: Clinical and therapeutical implications. *Curr Med Chem* 2009;16:940-952.

9. Goh SY, Cooper ME. Clinical review: The role of advanced glycation end products in progression and complications of diabetes. *J Clin Endocrinol Metab* 2008;93:1143-1152.

10. Bierhaus A, Hofmann MA, Ziegler R, Nawroth PP. Ages and their interaction with age-receptors in vascular disease and diabetes mellitus. I. The age concept. *Cardiovasc Res* 1998;37:586-600.

11. Opitz B, van Laak V, Eitel J, Suttorp N. Innate immune recognition in infectious and non-infectious diseases of the lung. *Am J Respir Crit Care Med*.

12. Lin L. Rage on the toll road? *Cell Mol Immunol* 2006;3:351-358.

13. Lanati N, Emanuele E, Brondino N, Geroldi D. Soluble rage-modulating drugs: State-of-the-art and future perspectives for targeting vascular inflammation. *Curr Vasc Pharmacol*;8:86-92.

14. Vazzana N, Santilli F, Cuccurullo C, Davi G. Soluble forms of rage in internal medicine. *Intern Emerg Med* 2009.

15. Schraml P, Bendik I, Ludwig CU. Differential messenger rna and protein expression of the receptor for advanced glycosylated end products in normal lung and non-small cell lung carcinoma. *Cancer Res* 1997;57:3669-3671.

16. Donato R. Intracellular and extracellular roles of s100 proteins. *Microsc Res Tech* 2003;60:540-551.

17. Bartling B, Hofmann HS, Weigle B, Silber RE, Simm A. Down-regulation of the receptor for advanced glycation end-products (rage) supports non-small cell lung carcinoma. *Carcinogenesis* 2005;26:293-301.

18. Hori O, Brett J, Slattery T, Cao R, Zhang J, Chen JX, Nagashima M, Lundh ER, Vijay S, Nitecki D, et al. The receptor for advanced glycation end products (rage) is a cellular binding site for amphoterin. Mediation of neurite outgrowth and co-expression of rage and amphoterin in the developing nervous system. *J Biol Chem* 1995;270:25752-25761.

19. Brett J, Schmidt AM, Yan SD, Zou YS, Weidman E, Pinsky D, Nowygrod R, Neeper M, Przysiecki C, Shaw A, et al. Survey of the distribution of a newly characterized receptor for advanced glycation end products in tissues. *Am J Pathol* 1993;143:1699-1712.

20. Matsuse T, Ohga E, Teramoto S, Fukayama M, Nagai R, Horiuchi S, Ouchi Y. Immunohistochemical localisation of advanced glycation end products in pulmonary fibrosis. *J Clin Pathol* 1998;51:515-519.

21. Bucciarelli LG, Wendt T, Rong L, Lalla E, Hofmann MA, Goova MT, Taguchi A, Yan SF, Yan SD, Stern DM, Schmidt AM. Rage is a multiligand receptor of the immunoglobulin superfamily: Implications for homeostasis and chronic disease. *Cell Mol Life Sci* 2002;59:1117-1128.

22. Geroldi D, Falcone C, Emanuele E. Soluble receptor for advanced glycation end products: From disease marker to potential therapeutic target. *Curr Med Chem* 2006;13:1971-1978.

23. Mukherjee TK, Mukhopadhyay S, Hoidal JR. Implication of receptor for advanced glycation end product (rage) in pulmonary health and pathophysiology. *Respir Physiol Neurobiol* 2008;162:210-215.

24. Bernard GR, Artigas A, Brigham KL, Carlet J, Falke K, Hudson L, Lamy M, Legall JR, Morris A, Spragg R. The american-european consensus conference on ards. Definitions, mechanisms, relevant outcomes, and clinical trial coordination. *Am J Respir Crit Care Med* 1994;149:818-824.
