# Supplementary material for: Soluble Forms and Ligands of the Receptor for Advanced Glycation End-Products in Patients with Acute Respiratory Distress Syndrome: An Observational Prospective Study
Source: PLoS One. 2015 Aug 14;10(8):e0135857. doi: 10.1371/journal.pone.0135857 (PMC4537285; doi:10.1371/journal.pone.0135857)
Supplement: S1 Table — (DOCX) [file pone.0135857.s003.docx]

| **Biological marker** | **ARDS**  **group** |  | **CONTROL**  **group** | **P** |
| --- | --- | --- | --- | --- |
| **sRAGE (pg/ml)**  *Arterial sRAGE*  D0H0  D0H4  D3  D6  *Central venous sRAGE*  D0H0  D0H4  D3  D6  *Alveolar sRAGE*  D0H0  D0H4  D3  D6  **esRAGE (ng/ml)**  *Arterial esRAGE*  D0H0  D0H4  D3  D6  *Central venous esRAGE*  D0H0  D0H4  D3  D6  *Alveolar esRAGE*  D0H0  D0H4  D3  D6  **HMGB1 (ng/ml)**  *Arterial HMGB1*  D0H0  D0H4  D3  D6  *Central venous HMGB1*  D0H0  D0H4  D3  D6  *Alveolar HMGB1*  D0H0  D0H4  D3  D6  **S100A12 (pg/ml)**  *Arterial S100A12*  D0H0  D0H4  D3  D6  *Central venous S100A12*  D0H0  D0H4  D3  D6  *Alveolar S100A12*  D0H0  D0H4  D3  D6  **AGEs (μg/ml)**  *Arterial AGEs*  D0H0  D0H4  D3  D6  *Central venous AGEs*  D0H0  D0H4  D3  D6  *Alveolar AGEs*  D0H0  D0H4  D3  D6 | 4812 [3211-6770]  3231 [2217-4472]  2482 [1274-2941]  893 [686-1876]  3890 [2624-5320]  2633 [2189-4283]  2154 [1363-3941]  1198 [747-1746]  36466 [12426-127398]  19040 [4689-58529]  2780 [1069-18261]  3352 [945-8826]  11 [4-24]  4 [2-11]  8 [1-18]  2 [1-10]  14 [6-28]  7 [3-18]  10 [1-21]  5 [1-13]  47 [31-76]  37 [12-60]  31 [21-51]  29 [6-51]  105 [3-345]  106 [3-347]  106 [3-347]  121 [3-346]  105 [3-344]  105 [3-346]  105 [3-345]  121 [3-346]  157 [19-350]  106 [4-346]  122 [4-345]  190 [3-360]  256680 [128400-547960]  241050 [124000-354800]  237740 [89403-392650]  128800 [91317-309399]  258771 [142800-572000]  256550 [106000-398215]  295400 [103573-452240]  142034 [10939-233520]  118177 [57000-563895]  56239 [30257-382642]  132067 [53759-453602]  152500 [82552-210091]  0.16 [0.03-1.32]  0.15 [0.02-1.32]  0.18 [0.06-0.73]  0.08 [0.02-0.16]  0.16 [0.07-1.46]  0.15 [0.14]  0.28 [0.07-1]  0.12 [0.03-0.22]  0.11 [0.05-2.1]  0.09 [0.04-0.72]  0.19 [0.05-2.7]  0.06 [0.03-3.8] |  | 938 [598-1096]  1013 [732-1190]  784 [694-988]  628 [472-858]  1062 [753-1361]  1116 [858-1341]  815 [728-1026]  643 [542-893]  935 [780-1471]  1178 [842-3587]  624 [316-906]  634 [305-1217]  104 [1-345]  104 [1-345]  103 [1-343]  119 [1-344]  104 [1-346]  103 [1-343]  119 [2-349]  119 [1-344]  103 [1-343]  103 [1-343]  131 [12-346]  187 [1-358]  8 [5-28]  8 [5-14]  4 [3-7]  5 [4-15]  7 [5-20]  7 [5-14]  4 [3-8]  5 [4-10]  20 [9-88]  36 [12-80]  35 [6-91]  82 [6-98]  38382 [27111-58412]  5941 [956-32417]  2683 [1421-12693]  10113 [8485-69296]  53217 [50017-64030]  27420 [8960-52877]  2438 [1066-24388]  15622 [11957-63644]  6242 [5758-59265]  24282 [4509-48447]  8569 [2805-18890]  8242 [7394-8941]  102 [0.06-342]  102 [0.08-342]  103 [0.08-343]  118 [0.27-344]  102 [0.08-342]  102 [0.12-342]  102 [0.09-342]  118 [0.4-344]  103 [0.16-342]  118 [0.09-345]  118 [0.08-344]  187 [2.6-361] | <10^-3^  <10^-3^  10^-3^  0.02  <10^-3^  <10^-3^  <10^-3^  0.01  <10^-3^  <10^-3^  <10^-3^  0.002  0.05  0.003  0.01  0.002  0.06  0.009  0.01  0.003  0.4  0.07  0.08  0.08  0.04  0.03  0.001  0.005  0.04  0.02  0.006  0.006  0.004  0.1  0.1  0.07  <10^-3^  <10^-3^  <10^-3^  <10^-3^  <10^-3^  <10^-3^  <10^-3^  <10^-3^  <10^-3^  <10^-3^  <10^-3^  <10^-3^  0.002  0.004  0.001  <10^-3^  0.001  0.003  0.002  <10^-3^  <10^-3^  <10^-3^  <10^-3^  <10^-3^ |
|  |  |  |  |  |

**Table S1. Levels of sRAGE, esRAGE, HMGB1, S100A12 and AGEs (median [Interquartile] at various study timepoints in patients with or without acute respiratory distress syndrome.** *D0H0 : baseline ; D0H4 : 4 hours after inclusion ; D3 : day 3 ; D6 : day 6.*
